# Mechanisms of Salt Tolerance and Molecular Breeding of Salt-Tolerant Ornamental Plants

**DOI:** 10.3389/fpls.2022.854116

**Published:** 2022-04-27

**Authors:** Jianrong Guo, Changdan Shan, Yifan Zhang, Xinlei Wang, Huaying Tian, Guoliang Han, Yi Zhang, Baoshan Wang

**Affiliations:** ^1^Shandong Provincial Key Laboratory of Plant Stress, College of Life Science, Shandong Normal University, Ji’nan, China; ^2^College of Forestry Engineering, Shandong Agriculture and Engineering University, Ji’nan, China

**Keywords:** ornamental plants, salinized soil, salt tolerance, breeding, value

## Abstract

As the area of salinized soils increases, and freshwater becomes more scarcer worldwide, an urgent measure for agricultural production is to use salinized land and conserve freshwater resources. Ornamental flowering plants, such as carnations, roses, chrysanthemums, and gerberas, are found around the world and have high economic, ornamental, ecological, and edible value. It is therefore prudent to improve the salt tolerance of these important horticultural crops. Here, we summarize the salt-adaptive mechanisms, genes, and molecular breeding of ornamental flowering crops. We also review the genome editing technologies that provide us with the means to obtain novel varieties with high salinity tolerance and improved utility value, and discuss future directions of research into ornamental plants like salt exclusion mechanism. We considered that the salt exclusion mechanism in ornamental flowering plants, the acquisition of flowers with high quality and novel color under salinity condition through gene editing techniques should be focused on for the future research.

## Introduction

Degradation of soils caused by salinization, a major abiotic stress factor, is an increasing limitation of arable land. Almost 10% of all soils and 50% of the irrigated land worldwide are affected by salinity (Guo et al., 2015, 2018; Wang et al., 2015, 2018; Song et al., 2016, 2017). Salinized soils limit the growth, development, and survival of plants that grow in such environments. Salinity poses a serious threat to food production and security (Munns and Tester, 2008; Abdullakasim et al., 2018). As the human population grows and urbanization increases, the area of land suitable for cultivation is decreasing. Improving living standards also mean increased demand for habitable land. Planting salt-tolerant flowering plants has become a feasible and sustainable strategy by which to use saline land without incurring competition for soils for food cultivation. Therefore, there is much interest in breeding high-value salt-tolerant ornamental flowering varieties, and to expand the usage of underutilized ornamental species for exploitation of saline soils.

Excess salinity in soils directly reduces the water potential (become more negative) around the roots, making it difficult for root cells to extract water and leading to water deficit (Chinnusamy et al., 2005; Ulczycka-Walorska et al., 2020; Wani et al., 2020; Zheng et al., 2021). Thus, to survive in such environments, plants must reduce the water potential of their own cells (Shabala et al., 2015). When salt ions accumulate in plant cells, ionic toxicity occurs. Subsequently, secondary damage such as oxidative stress and nutrient deficiencies can occur (Breś et al., 2016; Van Zelm et al., 2020). Some salt-sensitive plants may display stunted growth or even die because of damage caused by salinity. However, salt-tolerant plants can initiate various protective mechanisms that allow them to grow in saline environments; for example, changes in gene expression and regulation allow these plants to adapt their morphology, physiology, and biochemistry in response to salinity (Bai et al., 2018; Zandalinas et al., 2018; Guo et al., 2019a; Qi et al., 2020; Zhao et al., 2020). Additionally, the damage to plants caused by salinity depends on the species and environmental factors.

Flowering plants have many important roles for humans. They can be used as ornamental growing plants, as cut flowers, for environmental greening, as medicine, as fruits and vegetables. In addition, the flowering time of many plants can be changed to meet the option or the market needs. Recent reviews have explored the biodiversity of edible flowers (Boutigny et al., 2020), flower color regulation (Zhao and Tao, 2015), and breeding of mutant ornamental flowering plants (Anne and Lim, 2020). However, a review of the mechanisms of salt tolerance in ornamental flowering plants and of strategies to produce salt-tolerant ornamental plants or breed new, salt-tolerant varieties is lacking. Salinity is particularly responsible for degrading the visual quality of ornamental flowers (Jaleel et al., 2008; Yang et al., 2019). Therefore, here we review the salt-tolerant mechanisms of ornamental flowering plants, and the selection and breeding of new salt-tolerant ornamental flowering varieties.

## Salt Tolerance Mechanisms of Ornamental Plants

The level of salt tolerance of ornamental plants depends on the species, their development, the level of salt stress, and environmental factors. If the ornamental plant could grow and survive at the salt level at or over 200 mM NaCl that could be considered as a halophyte, thus the ornamental plants can be divided in halophytic and non-halophytic ones according to their salt tolerant ability. For example, the salinity threshold was 400 mM NaCl of the halophytic *Limonium sinuatum* under laboratory conditions (Mi et al., 2021). Many research findings have indicated that the development of salt-tolerant ornamental flowers is a viable approach. Although the opening time of the first flower was delayed, marigold (*Tagetes erecta*) can be planted in saline fields (García-Caparrós et al., 2016; Hao et al., 2017). Some ornamental species, such as fuchsia (*Fuchsia hybrida*), coleus (*Solenostemon scutellarioides*), and begonia (*Begonia hiemalis*; Villarino and Mattson, 2011), can tolerate a certain concentration of NaCl (7.0–9.8 dS m^−1^) without demonstrable growth inhibition. No negative impact was detected in lily (*Lilium × elegans*) seedlings treated with a moderate concentration of salt (3 dS m^−1^). Furthermore, the effect of salinity on growth could be alleviated by applying K^+^ to the grown medium (Ayad et al., 2019).

**Figure 1:**
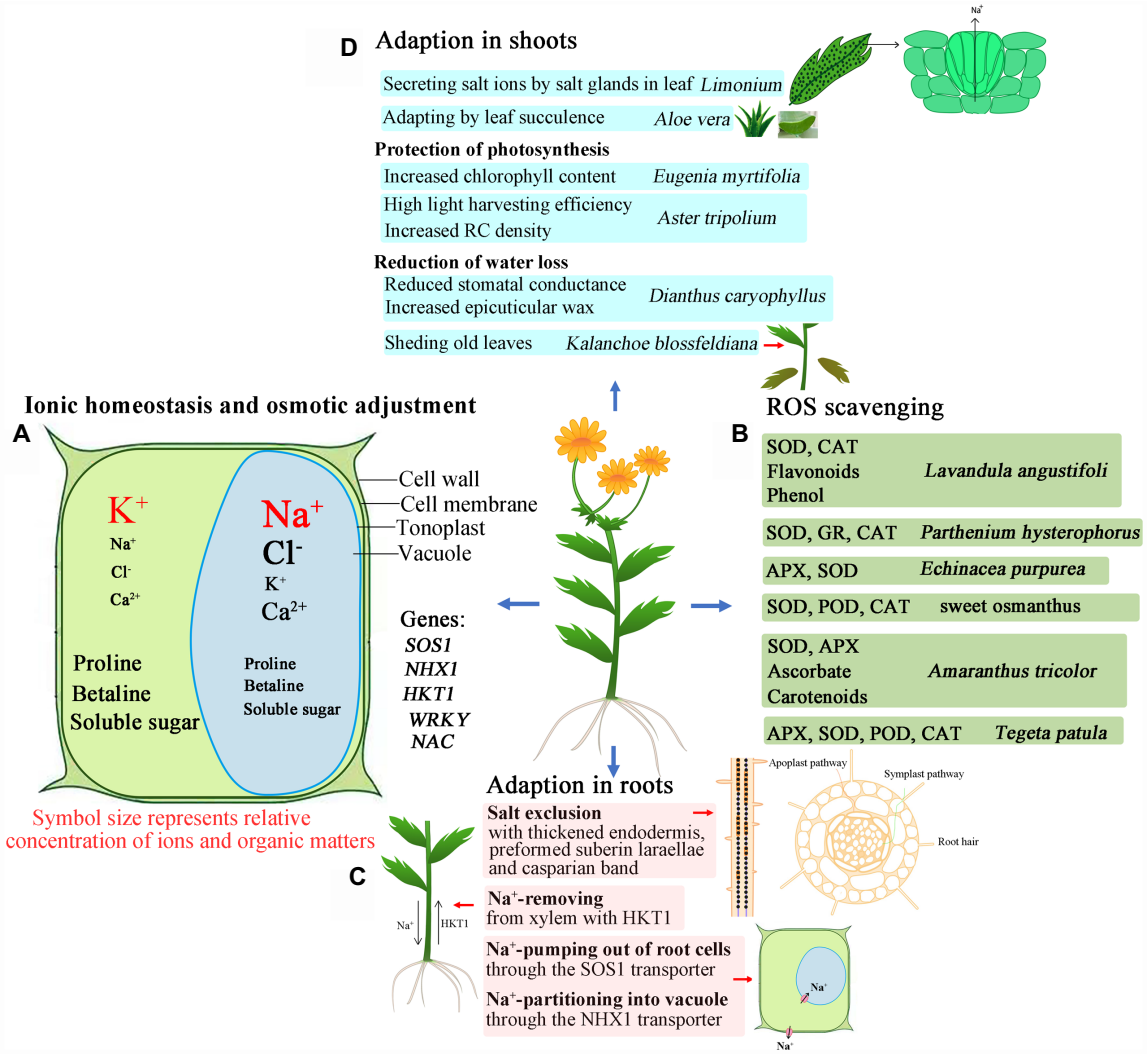
Graphical summary of the adaptation of ornamental plants to salinized environments. **(A)** Adaption through ionic homeostasis and osmotic adjustment; **(B)** Adaption through ROS scavenging; **(C)** Adaption through salt exclusion, removing and pumping out of cells in roots; **(D)** Adaption through salt secretion, leaf succulence, photosynthesis protection, and reduction of water loss in shoots. *SOS1*: Salt Overly Sensitive 1, e.g., *CcSOS1* gene from *Chrysanthemum crissum*; *NHX1*: vacuolar Na^+^/H^+^ antiporter, e.g., *IbNHX2* gene from *Ipomoea batatas*; *HKT1*: high-affinity K^+^ transporters, e.g., *AlHKT2:1* gene from *Aeluropus lagopoides*; WRKY: e.g., *DgWRKY4* genes of from *Dendronthema grandiform*; *NAC*: e.g., *DgNAC1* gene from *Dendronthema grandiform*; ROS: reactive oxygen species; SOD: superoxide dismutase; CAT: catalase; APX: ascorbate peroxidase; POD: peroxidase; GR: glutathione reductase; RC: reaction center. Symbol size represents relative content of ions and organic matter in plant cells.

Although there has been some research into the salt tolerance of ornamental plants, the underlying mechanisms are complex, and vary between ornamental plants. Therefore, detailed studies still need to be conducted to provide basis for future research and utilization of ornamental plants. The salt-tolerant mechanisms of ornamental plants are summarized in Figure 1, and the detailed experimental condition of plant species is tabulated in Table 1. These mechanisms include the regulation of osmotic balance under the osmotic stress caused by high salinity; adjustment of ionic balance to avoid ionic toxicity; active oxygen scavenging to reduce oxidative damage; the exclusion of salt from the roots by enhancing apoplastic barriers (like *Chrysanthemum*), salt release *via* Na^+^/H^+^ antiporters (like *Salicornia*), and salt secretion by salt glands or salt bladders (like *Atriplex*); photosynthetic regulation to maintain high photosynthetic efficiency (like *Aster*); limiting water loss by closing stomata, and thickening leaf surface wax (like *Dianthus*).

**Table 1:** The detailed experimental conditions of the ornamental flowering plants exposed to salt stress.

Species	Duration and extent of NaCl stress	Main phenotype	References
*Gerbera jamesonii*	30 mM/57 days	Mitigated phenotypes in leaves and flowers	Gómez Bellot et al., 2018
*Narcissus* sp. (L.)	150 mM/5–6 weeks	Maintained a steady K^+^/Na^+^ ratio	Veatch-Blohm et al., 2014
*Lobelia erinus*	30–50 mM/60 days	Reduced growth and biomass with no effect on flower production	Escalona et al., 2013
*Viola x wittrockiana* Gams.	5–7 g L^−1^/8 weeks	No effect on flower production with reduced growth of plant	Pušić et al., 2019
*Catharanthus roseus*	80 mM/30 days	Inhibited growth and the activities of antioxidant enzymes	Jaleel et al., 2008
*Limonium*	More than 200 mM/60 days	Grown well with salt excreting in leaves	Souid et al., 2016; Mi et al., 2021
*Aster tripolium* L.	250 mM/2 weeks	High light harvesting efficiencies and low energy dissipation rates	Duarte et al., 2017
*Callistemon citrinus*	4 dS m^−1^/56 weeks	Greater Na^+^ storage	Álvarez and Sanchez-Blanco, 2014
*Portulaca*	400 mM/5 weeks	Leaf succulence with high ionic accumulation in the vacuoles	Borsai et al., 2020
*Iris germanica*	140 mM/28 days	Lower Na^+^ content in rhizomes than in leaves or roots under saline conditions	Zhao et al., 2021
*Tagetes erecta*	4.5 or 7.5 dS m^−1^/60 days	Delayed the opening time of the first flower	García-Caparrós et al., 2016; Hao et al., 2017
*Dianthus caryophyllus*	200 mM/15 days	Reduced stomatal conductance and increased epicuticular wax	Kwon et al., 2019
*Chrysanthemum morifolium*	0.4–2.0%/1 week	Increased photosynthetic pigments with a better seedling growth	Vanlalruati et al., 2019
*Fuchsia hybrida*	7.0–9.8 dS m^−1^/5 weeks	With no demonstrable growth inhibition	Villarino and Mattson, 2011
*Solenostemon scutellarioides*	7.0–9.8 dS m^−1^/5 weeks	With no demonstrable growth inhibition	Villarino and Mattson, 2011
*Begonia hiemalis*	7.0–9.8 dS m^−1^/5 weeks	With no demonstrable growth inhibition	Villarino and Mattson, 2011
*Hyacinthus orientalis* L.	6.1–8.6 g dm^−3^/12 weeks	Accumulated more proline	Ulczycka-Walorska et al., 2020
*Pelargonium hortorum* L.H. Bailey	Lower than 1.976 g L^−1^/77 days	Accumulated more proline and anthocyanin	Breś et al., 2016
*Osmanthus fragrans* (Thunb.) Lour.	40–120 mM/3 days	Accumulated more proline	Geng et al., 2019
*Nerium oleander* L.	80 mM/15–30 days	Increased activities of SOD and CAT	Kumar et al., 2017
*Lavandula angustifoli* Mill.	300 mM/30 days	Increased activities of SOD and CAT	Szekely-Varga et al., 2020
*Parthenium hysterophorus*	160 mM/10 days	Increased activities of SOD, GR, CAT and contents of proline, Ascorbate, and glutathione	Ahmad et al., 2017
*Eugenia myrtifolia* L.	88 mM/30 days	Increased activities of SOD	Acosta-Motos et al., 2015
*Tagetes patula* L.	100 mM/10 days	Increased activities of antioxidant, and contents of polyphenol, carotenoid	Chrysargyris et al., 2018
*Echinacea purpurea*	50–100 mM/2 weeks	Increased activities of SOD and APX	Sabra et al., 2012
*Amaranthus tricolor*	50–100 mM/24 days	Increased activities of SOD, APX and ascorbate, carotenoids	Sarker and Oba, 2020
*Portulaca grandiflora*	400 mM/5 weeks	Enhanced salt tolerance	Borsai et al., 2020
*Iris halophila* (Pall.)	400 mM/5 weeks	Enhanced salt tolerance	Borsai et al., 2020
*Eragrostis spectabilis* (Pursh) Steud.	5.0 or 10.0 dS m^−1^/65 days	High visual quality was maintained	Wang et al., 2019c
*Viburnum lucidum* L.	200 mM/103 days	Higher chlorophyll and K^+^ content when applying of exogenous GB or proline	Cirillo et al., 2016
*Callistemon citrinus* Stapf	200 mM/103 days	Higher chlorophyll and K^+^ content when applying of exogenous GB or proline	Cirillo et al., 2016
*Aloe vera* (L.)	2.0–7.5 dS m^−1^/60 days	Higher ions in roots than in shoots	García-Caparrós et al., 2016
*Kalanchoe blossfeldiana* Poelln.	2.0–7.5 dS m^−1^/60 days	Reduced Na^+^ when older leaves are shed	García-Caparrós et al., 2016
*Gazania splendens* Lem sp.	2.0–7.5 dS m^−1^/60 days	Increased the succulence index with more Na^+^ and Cl^−^ in roots	García-Caparrós et al., 2016
*Viburnum lucidum*	10–70 mM/120 days	Higher ions in roots than in shoots	Cassaniti et al., 2009
*Alternanthera bettzickiana* (Regel) G. Nicholson	40 dS m^−1^/20 days	Higher Na^+^ content in roots than shoots	Ali et al., 2012
*Callistemon laevis*	4 dS m^−1^/56 weeks	Higher Na^+^ content in roots than stems	Álvarez and Sanchez-Blanco, 2014
*Dendrobium* orchid	2–15 dS m^−1^/30 days	Higher Na^+^ and Cl^−^ content in roots than shoots	Abdullakasim et al., 2018

### Adaptation to Salinity by Osmotic Adjustment and Ionic Homeostasis

Like many other plants, the first threat faced by ornamental plants in a saline environment is the lowered external water potential caused by the presence of salt ions. This prevents plant roots from taking up water from the soil, and can even cause water to drain out of the cells (Feng et al., 2014a), thus reducing plants’ ability to grow and survive. Accumulation of organic and inorganic solutes in the root cell cytoplasm (Feng et al., 2015) reduces the water potential of the cells and ensures water uptake under salt stress conditions (Han et al., 2012; Shao et al., 2014). In the process, some small organic molecules (compatible solutes), such as proline, will synthesize and accumulate in the cytosol of plant cells as the osmotic adjustment substances to cope with the osmotic stress (El-Shawa et al., 2020). These are also used to protect the structure of cells and macromolecular substances. In some cases, exogenous application of compatible solutes can increase plant salt tolerance. For example, increased antioxidant capacity was observed in plants to which exogenous glycine betaine (GB) was applied, compared with those without (Roychoudhury and Banerjee, 2016). At the same time, inorganic ion accumulation is also the way in which most salt-tolerant plants, such as halophytes, to reduce the osmotic potential. In this process, ions such as Na^+^ and Cl^−^ mainly accumulate in the vacuole so they can be used for osmotic adjustment of the plant cell (Chen and Jiang, 2010). Accumulation of inorganic ions as a strategy that consumes less energy than synthesize organic substances.

In addition, more Na^+^ and Cl^−^ accumulated in the roots than in the shoots of *Dendrobium* orchid under saline conditions. This high Na^+^/K^+^ ratio in the roots contributed to osmotic adjustment (Abdullakasim et al., 2018). More proline accumulated in seedlings of sweet osmanthus [*Osmanthus fragrans* (Thunb.) Lour.], germinated from gamma-irradiated seeds than those of controls, thus the salt tolerance of the seedlings was increased by improving osmotic adjustment (Geng et al., 2019). Salinity reduced the leaf area and flower weight of marigold (*Calendula officinalis* L.), but increased the proline content in these plants. This increased proline content conferred salt tolerance to marigolds in saline conditions below 150 mM NaCl (Adamipour et al., 2019). Despite decreased seedling biomass, proline accumulated and was the main osmolyte responsible for the osmotic adjustment of ornamental plants in response to saline conditions (El-Shawa et al., 2020). In the ornamental crop *Hyacinthus orientalis* L., proline in the leaves increased under saline conditions of 6.1–8.6 g L^−1^. The ornamental qualities of these flowers also increased, indicating promise that this crop could be cultured in saline soils with concentrations of sodium chloride as was used in the laboratory culture (Ulczycka-Walorska et al., 2020).

Ionic homeostasis in plant cells is the basis for development and metabolism, especially the higher K^+^ and lower Na^+^ concentration in the cytosol of salt-tolerant plant cells (Blumwald, 2000). In salt sensitive plants under salt stress, ionic disturbance is very important, while salt-tolerant plants have a greater ability to maintain ionic balance (Guo et al., 2019a). With a similar charge and physiological characteristics, Na^+^ might compete with K^+^ for active sites of enzyme transporters. Thus, competition between the two ions leads to disruption of enzymatic function and biosynthesis (Zhu, 2003). For plants to grow and develop well under saline conditions, maintenance of a proper ratio of cytosolic K^+^/Na^+^ is required. This, along with the homeostasis of other ions, such as Ca^2+^, Mg^2+^ and Fe^2+^, is also a reliable indicator of the level of plants’ salt tolerance (Munns and Tester, 2008).

In salt-tolerant plants, ionic homeostasis might be maintained by excluding salt ions in soil through the roots by Salt Overly Sensitive 1 (SOS1) and apoplastic barriers (Hose et al., 2001; Meyer et al., 2009; Krishnamurthy et al., 2011; Song et al., 2012; Gao et al., 2016); thus fewer ions might be translocated to, or accumulate in, the aboveground parts of the plant, such as leaf cells, by compartmentalizing salt ions into the vacuole and reducing the ionic concentration in cytoplasm (Qiu et al., 2007). Therefore, restraining ionic transport to the shoots and compartmentalizing toxic ions in the vacuole are the important pathways to enhance plants’ salt tolerance in saline environments (Hasegawa, 2013). In the ornamental plant *Eugenia myrtifolia* L., more salt ions accumulated in roots than in leaves to perform normal physiological metabolism under salinity conditions (Acosta-Motos et al., 2017). In salt-tolerant chrysanthemum varieties, growth increased under saline conditions, together with increased K^+^, Ca^2+^ and Mg^2+^ content, and more efficient usage of N and P (Rahi and Singh, 2011). Results in marigold (*Tegeta patula*) indicated that salt tolerance could be improved by enhanced potassium application (Aboutalebi Jahromi and Hosseini Farahi, 2016).

For the ornamental plant gerbera (*Gerbera jamesonii Bolus* ex Hook. f., cv. “Forsa”), the presence of NaCl (such as 30 mM) could mitigate the phenotypes in leaves and flowers affected by boron (Gómez Bellot et al., 2018). A steady K^+^/Na^+^ ratio was maintained despite high Na^+^ accumulation in the leaves of daffodils [*Narcissus* sp. (L.) Amaryllidaceae] when treated with different salt concentrations—even under 150 mM NaCl conditions (Veatch-Blohm et al., 2014). While for the halophytic ornamental plant *Lobularia maritima* L., which growth was unaffected by 100 mM NaCl with the relative ionic balance in roots and leaves (Hsouna et al., 2020). Another halophytic ornamental plant *Sesuvium portulacastrum* displayed an optimal growth treated with moderate salinity with accumulating of large amounts of true halophyte and shows an optimal development under moderate salinity with a large amount of salt ions accumulating in the leaves (Nikalje et al., 2018; Ding et al., 2022). In *Callistemon laevis* treated with NaCl, little Na^+^ concentration was detected in the leaves, and more Na^+^ accumulated in the roots and stems, indicating that more salt ions were prevented from reaching the aboveground parts of the plant (Álvarez and Sanchez-Blanco, 2014). In ornamental grasses, such as *Eragrostis spectabilis* (Pursh) Steud., and *Panicum virgatum* L. “Northwind,” high visual quality was maintained when treated with NaCl, despite high concentrations of salt ions (Na^+^ and Cl^−^) accumulating in leaves (Wang et al., 2019c). In *Lobelia erinus*, plant growth and biomass were reduced with increased salinity, while salinity (30 or 50 mM NaCl) had no effect on flower production, and no toxic symptoms were observed on leaves, despite high accumulation of Na^+^ and Cl^−^ in leaf tissues. This indicates that *L. erinus* could be used for urban landscaping (Escalona et al., 2013). A similar result was observed in pansy (*Viola x wittrockiana* Gams.), which displayed salt tolerance at levels of 5 or 7 g L^−1^ NaCl (Pušić et al., 2019). Under salt stress, uptake of Ca^2+^, Mg^2+^, and Na^+^ in the shoots of marigold (*Tagetes erecta* L.) increased, indicating an increase in salt tolerance (Koksal et al., 2016). When *Callistemon citrinus* plants were subjected to salt stress, greater Na^+^ storage was detected, which played a role in maintaining plant quality, and indicating that *C. citrinus* could be cultured with 4 dS m^−1^ saline water (Álvarez and Sanchez-Blanco, 2014).

It is well known that high salinity decreases K^+^ content in plants. A saline environment reduced the growth of marigold, with decreased K^+^ content, while the Mg^2+^ content increased in the leaves under such conditions (Koksal et al., 2016). However, the K^+^ content, as well as the carotenoid content, in pelargonium (*Pelargonium hortorum* L.H. Bailey) leaves was not affected when plants were grown at salinities lower than 1.976 g L^−1^. Meanwhile, the increased anthocyanin and proline content in leaves might be important in ameliorating the adverse effects caused by salt stress (Breś et al., 2016).

Salt-tolerant species can maintain ion homeostasis during growth, and this is inseparable from the roles of membrane-bound ion transporters, such as Na^+^/H^+^ antiporters. Under salt stress conditions, the plasma membrane-located Na^+^/H^+^ antiporter SOS1, and the NHX antiporters on tonoplast membranes are vital in maintaining the cellular ion homeostasis by transporting excess Na^+^ out of the cells and into the vacuole (Munns and Tester, 2008; Yue et al., 2012). In the working model of the SOS system pathway, cellular Ca^2+^ signals were identified as being involved. Firstly, salt stress induced an increased concentration of Ca^2+^ in the cytosol, which is sensed by, and causes the activation of SOS3. SOS2 is then activated by activated SOS3, forming an SOS2–SOS3 complex. Thirdly, SOS1 is activated, which mediates the transport of excess Na^+^ out of the cells and maintains a relatively low Na^+^ concentration in the cytoplasm (Tang et al., 2015). A significant reduction in Na^+^ content, and a favorable K^+^/Na^+^ ratio, was detected in transgenic plants—especially in the youngest leaves—in *Chrysanthemum crassum* overexpressing the *CcSOS1* gene. Thus, the transgenic plants demonstrated higher salt tolerance, up to 200 mM NaCl (An et al., 2014).

NHX family transporters are localized to the tonoplast, they are widely considered as the players to sequestrate sodium (Na^+^) into vacuoles and to avert the cytoplasmic ion accumulation when plants exposed to salinity. In Arabidopsis, the expressional levels of *AtNHX1* and *AtNHX2* genes were upregulated under osmotic stress or by abscisic acid (ABA; Jiang et al., 2011). Overexpression of HtNHX1 and HtNHX2 transporters from *Helianthus tuberosus* improved the salt tolerance of rice (Zeng et al., 2018). Salt or drought tolerance was enhanced in transgenic sweet potato [*Ipomoea batatas* (L.) Lam.] with overexpression of the *IbNHX2* gene (Wang et al., 2016).

In view of this research on the ornamental flowering plants, salt-tolerant varieties seem to prefer to accumulate inorganic ions in the vacuole to lower cell water potential, and simultaneously synthesize some compatible solutes in the cytosol to balance the decline of vacuolar water potential. This strategy thereby ensures the growth of plants and their increasing salt tolerance. A reconstruction of osmotic and ion homeostasis pathways in a plant cell under saline stress is shown in Figure 1A.

### Adaptation to Salinity by Maintaining the Balance of Reactive Oxygen Species

Reactive oxygen species (ROS) are free radical molecules that are inevitably produced during plant metabolism. They have important roles in plant growth, development, and adaptation to stressors. Under normal conditions, ROS production and scavenging is kept in balance. However, when plants are subjected to adversity, such as salt or drought stress, more ROS are produced, and they will accumulate if production exceeds scavenging. Excess accumulation of ROS damages structural and metabolic plant processes (Pang et al., 2011). In salt-tolerant species, antioxidant capacity is enhanced to scavenge accumulated ROS, thus restoring the balance of ROS. Usually, ROS scavenging is implemented in two main ways: non-enzymatic and enzymatic mechanisms (Bowler et al., 1992; Mittler, 2002). Superoxide dismutase (SOD) and catalase (CAT) are usually considered the two most important antioxidant enzymes, but others, such as ascorbate peroxidase (APX), peroxidase (POD), and glutathione reductase (GR), are all employed to scavenge ROS in enzymatic reaction systems (Kozi, 1999). Furthermore, they work together to regulate the balance of ROS in plants.

When ROS levels are in equilibrium, membrane stability is maintained and normal metabolism occurs in plants (Li et al., 2012; Luo et al., 2013). Tolerance to other stresses, such as drought or heat, is also enhanced (Reddy et al., 2004). During ROS scavenging, enzymic amount and enzymic activity are strongly related to plants’ ROS scavenging ability. Overexpression of genes related to antioxidant enzymes could improve ROS scavenging, thus enhancing stress tolerance (Caverzan et al., 2016). In the ornamental plant *Lavandula angustifoli* Mill., antioxidant enzymes, including SOD and CAT, were activated under salt stress (even at 300 mM NaCl), with enhanced accumulation of flavonoids and phenolic substances, thus improving plants’ oxidation tolerance (Szekely-Varga et al., 2020). In salt-treated *Echinacea* species, *E. purpurea* displayed higher Na^+^ exclusion ability, and enhanced antioxidant activity of APX and SOD, but CAT activity was reduced and there was no change in GR activity (Sabra et al., 2012). The highly salt-tolerant ornamental plant, *Parthenium hysterophorus* could grow in a concentration as high as 160 mM NaCl. It also displayed increased proline, ascorbate, and glutathione contents, and elevated activity of enzymatic antioxidants, such as SOD, GR, and CAT (Ahmad et al., 2017). With the high salt tolerance (800 mM NaCl) of the ornamental plant *Nerium oleander* L., the activities of SOD and CAT were increased in the leaves and roots, even though more salt ions accumulated in these organs (Kumar et al., 2017). In tagetes (*Tagetes patula* L.), polyphenol and carotenoid contents, and antioxidant activities increased under 100 mM NaCl, and there was high accumulation of N, P, Zn, and Na in the flowers (Chrysargyris et al., 2018). In *Eugenia myrtifolia* L., SOD content and activity in the leaves increased despite reduced APX content and activity under saline conditions, and lower Na^+^ in the leaves than roots ensured normal photosynthesis (Acosta-Motos et al., 2015). The antioxidant ability of plants could also be enhanced by gene mutation. The activities of SOD, CAT, and POD were significantly increased in seedlings of sweet osmanthus germinated from gamma-irradiated seeds compared with controls; seedlings’ salt tolerance was improved by regulating ROS balance (Geng et al., 2019). When treated with 80 mM NaCl, growth and the activities of antioxidant enzymes of *Catharanthus roseus* plants were inhibited. However, the salt tolerance of the seedlings was enhanced, with increased plant weight, SOD, and POD activities when plants were grown with NaCl and propiconazole, except leaf area and stem length decreased (Jaleel et al., 2008).

Salt tolerance is different even in different varieties of the same species. In *Amaranthus tricolor*, ascorbate, carotenoids (non-enzymatic antioxidant), and SOD and APX (antioxidant enzymes) activities were increased in the salt-tolerant varieties compared with the salt-sensitive ones, indicating that increased ROS quenching ability conferred higher tolerance (Sarker and Oba, 2020). The activities of APX, SOD, POD and CAT in marigold were enhanced when plants were grown under conditions below 150 mM NaCl, then decreased at 200 mM NaCl (Adamipour et al., 2019), indicating that marigold could be developed for cultivation on saline lands.

In saline environments, large amounts of ROS accumulate in plants, resulting in ROS imbalance. Salt-tolerant ornamental varieties usually show improved ROS scavenging ability, both from enzymatic and non-enzymatic reaction processes, so this could be considered an indicator of high salt tolerance in ornamental flowering plants (Figure 1B).

### Adaptation to Salinity by Root Salt Exclusion

Another measure that plants employ to reduce cellular ion content is to reduce the uptake of salt ions in the roots, or to excrete some absorbed salt ions out of the plant (Figure 1C). Thus, a relatively higher Na^+^ content will be detected in roots than in shoots (Abdullakasim et al., 2018). The same was found under cadmium stress (Qi et al., 2020). Salt tolerance was conferred by a special structure, such as thickened apoplastic barriers (Krishnamurthy et al., 2020). In some ornamental plants, such as *Escallonia rubra*, *Cestrum fasciculatum*, *Viburnum lucidum* (Cassaniti et al., 2009), and *Aloe vera* (L.; García-Caparrós et al., 2016), the mechanism employed to respond to salinity correlated with higher accumulation of ions in roots than in shoots. Na^+^ content in rhizomes of *Iris germanica* was usually lower than in leaves or roots under saline conditions, which may indicate a salt stress adaptation mechanism (Zhao et al., 2021). Perhaps, in such plants under saline conditions, the greater accumulation of salt ions in roots is associated with structural changes of roots related to the apoplastic exodermal barriers, such as the thickened endothelium, preformed suberin lamellae and Casparian bands (Andersen et al., 2015). In *Opisthopappus taihangensis* (Ling) Shih and *Iris germanica* (Meyer et al., 2009; Yang et al., 2020), salt ion uptake was reduced in root cells. Additionally, the absorbed ions in root cells could also be excreted out of the roots cells through antiporters such as SOS1 (Song et al., 2012; Gao et al., 2016; Mohammadi et al., 2019), resulting in increased ion accumulation in roots and reduced salt ion transport to shoots. However, the relationship between salt ion accumulation and apoplastic barriers remains to be investigated in ornamental flowering plants.

As well as reducing salt ion uptake through structural features, absorbed salt ions can also be removed from xylem. The *HKT1* (high-affinity K^+^ transporters) gene plays vital roles in this process (Ali et al., 2016), and the HKT1 transporter can also restrict Na^+^ entry into plant roots (Rus et al., 2001). With the function of HKT1 localized in xylem parenchyma cells, Na^+^ flux to the shoot tip was actively reduced and excess Na^+^ accumulated in the root zone of plants. In Arabidopsis, AtHKT1 was proposed to recirculate Na^+^ in the phloem, reducing the allocation of Na^+^ in the shoot, and correspondingly increasing Na^+^ content in roots (Davenport et al., 2007).

Furthermore, absorbed Na^+^ can also be excreted from root cells through transporters such as SOS1, which are localized in the plasma membrane of root cells (Shi et al., 2000; Oh et al., 2009), like the CcSOS1 transporter in chrysanthemum (Song et al., 2012; Gao et al., 2016). Perhaps, in ornamental flowering plants, the SOS1 transporters also play a vital role in pumping Na^+^ out of root cells.

Finally, absorbed salt ions (especially Na^+^) can be partitioned into the vacuole *via* transporters such as NHX1, which is localized in the tonoplast. On the one hand, vacuoles act as the ultimate Na^+^ sinks to reduce Na^+^ accumulation in cytoplasm. On the other hand, the water potential of root cells is also reduced to ensure water uptake in saline environments (Liang et al., 2015; Guo et al., 2020).

### Adaptation to Salinity by Shoots

Under saline conditions, the response mechanisms triggered are different in different ornamental species. In some ornamental species with salt secreting structures like salt glands or salt bladders, especially *Limonium* species (Feng et al., 2014b; Leng et al., 2018; Lu et al., 2020; Mi et al., 2021), excess salt ions transported to aboveground parts of the plant are also excreted outside of the plant body through salt glands (García-Caparrós et al., 2016). Limonium species have remarkable salt tolerance thanks to their typical salt excreting salt glands in the leaves; they can grow and develop well in environments containing NaCl concentrations of more than 200 mM NaCl (Souid et al., 2016; Mi et al., 2021). Besides excreting excess salt ions out of the leaves, some of the absorbed salt ions are regionalized; they accumulate in vacuoles and regulate the osmotic potential of leaf cells (González-Orenga et al., 2021). Therefore, more ornamental flowering plants that possess these leaf structures, and which also have high value, should be exploited further.

Some ornamental plants, such as *Portulaca* (Borsai et al., 2020), as well as *Aloe vera* (L.), exhibit leaf succulence when they grow in saline or drought environments (García-Caparrós et al., 2016). In response to salt stress, large amounts of ions were accumulated in the vacuoles of leaf cells in such plants, and the leaf succulence was subsequently displayed higher ability to cope with the salt stress. And the specific anatomical adaptation to salinity in halophytic ornamental flowering plants is summarized in Supplementary Figure S1.

To respond to saline conditions, ornamental plants have evolved many other adaptations; for example, altering leaf function, reducing chlorophyll content, and reducing stomatal size and aperture (García-Caparrós and Lao, 2018). In carnation (*Dianthus caryophyllus*), salt tolerance was mainly associated with the reduction of water loss by reducing stomatal conductance and increasing epicuticular wax (Kwon et al., 2019). *Chrysanthemum morifolium* treated with different levels of salinity displayed salt tolerance, with increased photosynthetic pigments, and a better seedling growth (Vanlalruati et al., 2019). Photosynthetic pigments are vital in photosynthesis. Under stress, inhibited photosynthetic efficiency in plants was related to reduce chlorophyll content and damaged photosynthetic systems (Wu et al., 2016). Some plants showed higher tolerance to salinity in photosynthetic processes. For example, low energy dissipation rates and high light harvesting efficiencies were detected in *Aster tripolium* L. plants exposed to even 250 mM NaCl, with increased density of reaction centers (RC), enhanced electron transport ability, and lower energy dissipation rates (Duarte et al., 2017). In the ornamental plant *Eugenia myrtifolia* L., when treated with salinity, the normal photosynthetic system was protected by limiting salt ion accumulation in leaves, as well as by increasing chlorophyll content (Acosta-Motos et al., 2017). The salt tolerance of *Aloe vera* associated with high efficiency of photosystem II and ROS scavenging was enhanced by exogenous application of proline (Nakhaie et al., 2020). With the application of exogenous GB or proline, higher chlorophyll and K^+^ content was detected in *Viburnum lucidum* L. and *Callistemon citrinus* Stapf (Cirillo et al., 2016), as well as calendula (*Calendula officinalis* L.) plants (Soroori et al., 2021). Proline content and the stability of the cellular membrane were increased by exogenous application of ascorbic acid on calendula plants under salinity stress (Azizi et al., 2021).

The salt tolerance of ornamental flowering plants could also be enhanced, and the salt content reduced, by shedding old leaves. For example, older leaves are shed to reduce Na^+^ content in *Kalanchoe blossfeldiana* Poelln. In *Gazania splendens* Lem sp., multiple strategies are employed, such as accumulating the majority of Na^+^ and Cl^−^ in the roots, secreting salt ions out of leaves through salt glands, increasing the succulence index of living leaf tissue, and shedding old leaves (García-Caparrós et al., 2016). Thus, *G. splendens* is suitable for planting in saline environments. The adaptive strategies of shoots are summarized in Figure 1D.

## Molecular Breeding For Improvement of Salt Tolerance in Ornamental Plants

Salinity is one of the most important adverse factors limiting agricultural production. Cultivating salt-tolerant ornamental crops or flowers is considered to be an effective way of utilizing saline soils. Therefore, selecting and breeding salt-tolerant ornamental flowering crops is a first and important step for the sustainable development of saline land. Many salt-tolerant plants can survive in heavily saline soils, and current research demonstrates the benefit of selecting and breeding species that are both ornamental and highly salt tolerant. And new varieties with higher salt tolerance could be obtained through gene mutation, such as *via* gamma irradiation. Seedlings with enhanced salt tolerance, enhanced osmotic regulation ability, and greater ROS scavenging capacity were obtained *via* gamma irradiation in sweet osmanthus (Geng et al., 2019).

To survive in a saline environment, plants—on the one hand—reduce the translocation of toxic ions to aboveground parts of the plant by draining salt ions to the root extracellular spaces. On the other hand, they accumulate excess salt ions into the vacuoles. Thus, the cytoplasmic ion content is maintained in controlled levels, the osmotic potential of plant cells is reduced, and normal water uptake and physiological metabolism can occur (Chen et al., 2010; Song and Wang, 2015). In this process, many genes are upregulated to ensure recruitment of proteins that participate in ion uptake, translocation, and redistribution. This, in turn, ensures that salt ion concentrations in plant cells are reduced to a minimum (Yang et al., 2017). If plants can successfully redistribute ions, the plant will survive; if the plant cannot successfully redistribute salt ions well, then plant growth will be inhibited, or the plant might even die, once the salt content exceeds the plant’s level of tolerance. Therefore, the ability of a plant to retain K^+^ and balance Na^+^ in the cytoplasm is one of the most important indicators of salt tolerance in plants. Research finds that the ability to retain K^+^ is particularly critical (Reddy et al., 2004; Caverzan et al., 2016; Wang et al., 2019b,c). Maintenance of intracellular ionic homeostasis in *Arabidopsis thaliana* depends primarily on *AtNHX1* (an Na^+^/H^+^ antiporter gene located in the tonoplast) and *AtSOS1* (an Na^+^/H^+^ antiporter gene located in plasma membrane; Zhu, 2003). Furthermore, the mechanism responsible for regulating salt ions in most plants is the same as in *A. thaliana*. Interestingly, species with high salt tolerance, such as halophytes, can better adjust or compartmentalize ions than Arabidopsis (Song, 2009; Guo et al., 2018, 2019a). Perhaps, higher expressional levels of related genes are activated in such plants. As in Arabidopsis, increased Na^+^ efflux and reduced intracellular Na^+^ content was detected in transgenic plants overexpressing *AtSOS1* and *AtHKT1* genes (Wang et al., 2014, 2019a). In the important ornamental plant *Chrysanthemum crissum*, salt tolerance in *CcSOS1* over-expressing plants was enhanced, with a significant reduction in Na^+^ content and a favorable K^+^/Na^+^ ratio. This plant could grow under 200 mM NaCl conditions (An et al., 2014). Overexpression of *CmPIP1* and *CmPIP2* genes from *Chrysanthemum morifolium* in transgenic chrysanthemum plants also resulted in salt tolerance (Zhang et al., 2019). And the salt tolerance was improved by overexpressing *IlNHX* from *Iris lacteal* (Guo et al., 2020). In the halophyte *Aeluropus lagopoides*, the promoter of the gene encoding the high-affinity potassium transporter AlHKT2;1 was involved in the response to salt stress in plants (Dave et al., 2021).

As well as the functional genes involved in ion transport during salt tolerance in ornamental plants, transcription factor genes are also involved in salt tolerance; this lays the foundation for further research into salt-tolerant mechanisms in ornamental plants. Novel varieties with high salt tolerance could be obtained using transgenic transcription factor genes. For example, the salt tolerance of transgenic chrysanthemum was enhanced by overexpressing the *DgNAC1* or *DgWRKY4* transcription factor genes from chrysanthemum (*Dendronthema grandiform*; Wang et al., 2017a,b). Salinity tolerance of Arabidopsis was conferred by overexpressing the *PSK1* gene (a homologous gene of S-phase kinase-associated protein1-like) from *Paeonia suffruticosa*, indicating that this gene is important for salt tolerance, together with the function of flowering in peony (Hao et al., 2017). The salt tolerance of chrysanthemum (*Dendronthema grandiform*) transgenic plants was improved with overexpression of the transcription factor gene *DgNAC1* (a salt responsive gene); higher activities of SOD, POD, and CAT were detected in the transgenic plants compared with wild type (Wang et al., 2017b). Thus, this could provide evidence for the molecular modification of salt tolerance in ornamental plants. Genes known to confer salt tolerance to ornamental flowering plants are summarized in Table 2.

**Table 2:** Genes involved in salt tolerance of salt-tolerant ornamental flowering plants.

Gene name	Species	Accession number	Probable function	References
*LfSOS1*	*Leptochloa fusca*	KC525946	Encoding a cytomembrane Na^+^/H^+^ antiporter that transport Na*^+^* out of the plant cells	Mohammadi et al., 2019
*CcSOS1*	*Chrysanthemum crissum*	AB439132	Encoding a cytomembrane Na^+^/H^+^ antiporter that transport Na*^+^* out of the plant cells	An et al., 2014
*CmSOS1*	*Chrysanthemum morifolium*	KP896477	Encoding a cytomembrane Na^+^/H^+^ antiporter that transport Na*^+^* out of the plant cells	Gao et al., 2016
*AjSOS1*	*Artemisia japonica*	KP896475	Encoding a cytomembrane Na^+^/H^+^ antiporter that transport Na*^+^* out of the plant cells	Gao et al., 2016
*CrcSOS1*	*Crossostephium chinense*	KP896476	Encoding a cytomembrane Na^+^/H^+^ antiporter that transport Na*^+^* out of the plant cells	Gao et al., 2016
*LfNHX1*	*Leptochloa fusca*	JF933902	Encoding a tonoplast Na^+^/H^+^ antiporter that transport Na*^+^* into vacuole	Mohammadi et al., 2019
*HtNHX1*	*Helianthus tuberosus*	EF159151	Encoding a tonoplast Na^+^/H^+^ antiporter that transport Na*^+^* into vacuole	Zeng et al., 2018
*HtNHX2*	*Helianthus tuberosus*	DQ343304	Encoding a tonoplast Na^+^/H^+^ antiporter that transport Na*^+^* into vacuole	Zeng et al., 2018
*IbNHX2*	*Ipomoea batatas*	JN688961	Encoding a tonoplast Na^+^/H^+^ antiporter that transport Na*^+^* into vacuole	Wang et al., 2016
*IlNHX*	*Iris lacteal*	AY730277	Encoding a tonoplast Na^+^/H^+^ antiporter that transport Na*^+^* into vacuole	Guo et al., 2020
*AlHKT2;1*	*Aeluropus lagopoides*	MW535306	Encoding a transporter recirculating Na^+^ from shoot to root	Dave et al., 2021
*PsPSK1*	*Paeonia suffruticosa*	FE529999	Encoding a kinase-associated protein involving in response to salt tolerance	Hao et al., 2017
*DgNAC1*	*Dendronthema grandiform*	HQ317452	NAC transcription factor gene that response to salt tolerance	Wang et al., 2017a
*DgWRKY4*	*Dendronthema grandiform*	KC615358	WRKY transcription factor gene that response to salt tolerance	Wang et al., 2017b
*CmPIP1*	*Chrysanthemum morifolium*	KJ489416	Encoding aquaporin involving in salt tolerance	Zhang et al., 2019
*CmPIP2*	*Chrysanthemum morifolium*	KJ756774	Encoding aquaporin involving in salt tolerance	Zhang et al., 2019

The ornamental flower *Alternanthera bettzickiana* (Regel) G. Nicholson grew under saline conditions, with less Na^+^ content in shoots than roots, even at 40 dS m^−1^, so was considered to be a potential halophyte (Ali et al., 2012). Plants with high salt tolerance, such as *Portulaca grandiflora* and *Iris halophila* (Pall.) plants, which exhibit high salt tolerance during the growth period (Borsai et al., 2020), could be used as greening species on saline soils. Selection and utilization of such species could enable further study of salt tolerance mechanisms at the molecular level (Liu et al., 2018). Systematic genomic analysis to identify salt tolerance-related genes or transcription factors will be of great significance in plant breeding (Wang et al., 2021). Many transcription factor genes and functional genes might serve as candidate genes for breeding salt-tolerant ornamental flowering plants.

## Acquisition of Novel Varieties by Genetic Modification

The advent of genome editing technology, especially the CRISPR editing system, has provided an efficient method to obtain improved flower varieties. The genome sequences of some ornamental crops are available, such as *Nankingense*, *Chrysanthemum*, *Rosa chinensis*, *Ipomoea nil*, *Petunia hybrid*, and *Dendrobium officinale*. These genomes will provide the basis for obtaining and developing new varieties of ornamental crops, such as early flowering varieties of orchid and chrysanthemum (Ahmad et al., 2021). Using CRISPR/Cas9 genome editing technology, more flower colors were obtained in *Lilium* (Yan et al., 2019). In *Torenia fournieri* L., CRISPR/Cas9 modification of a gene related to flavonoid synthesis resulted in flowers with a pale purple and almost white color (Nishihara et al., 2018). Flower longevity was extended in *Petunia hybrida* by knocking out the gene for 1-aminocyclopropane-1-carboxylate oxidase 1 (*ACO*), which reduced the production of ethylene (Xu et al., 2020). Altered flower color (influenced by biosynthesis of anthocyanin) was also obtained in *Ipomoea nil* (Japanese morning glory) using CRISPR/Cas9 (Watanabe et al., 2017).

**Figure 2:**
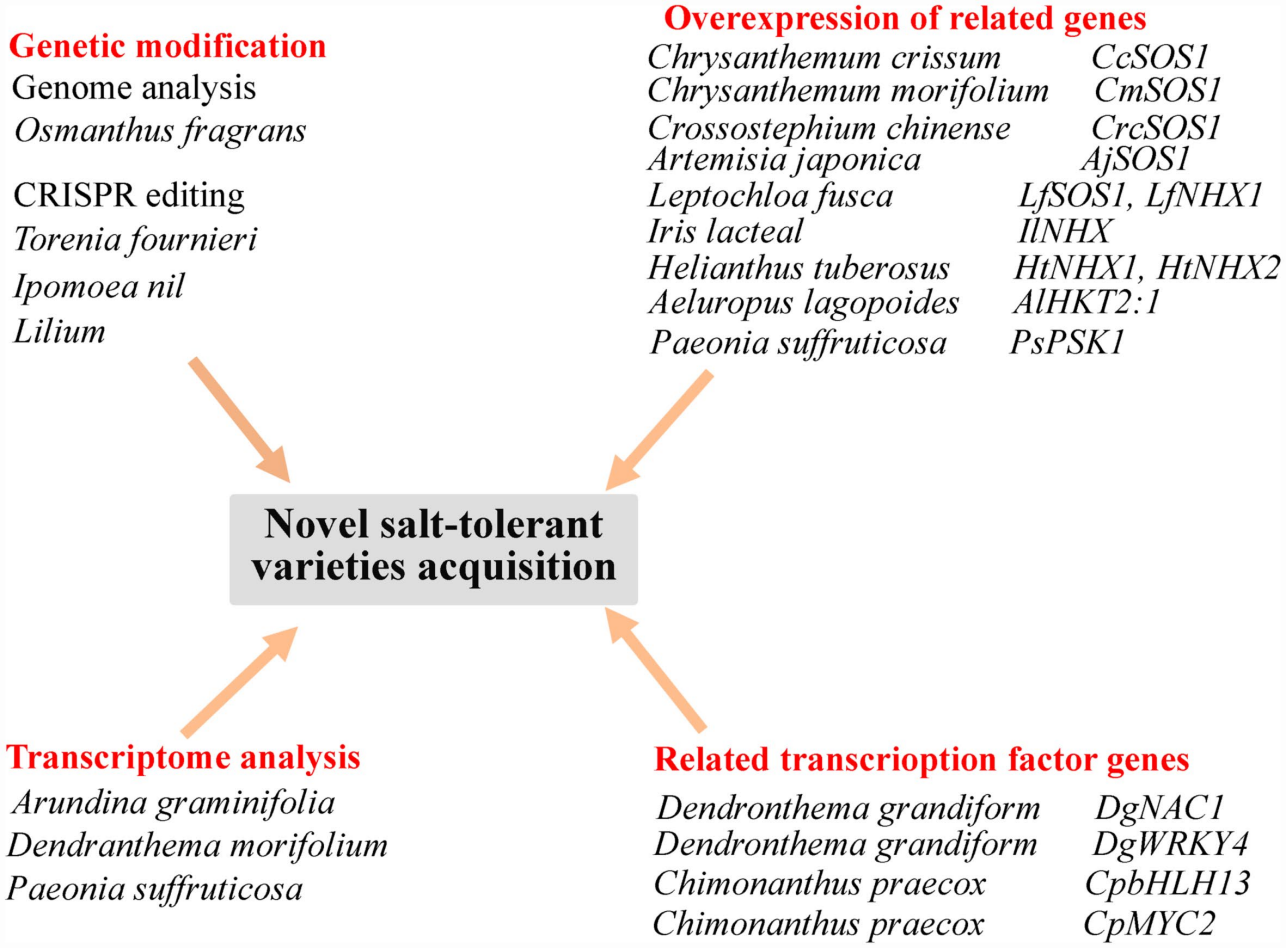
Summary of approaches for the acquisition of novel, salt-tolerant ornamental flowering plant varieties. *CcSOS1*, cytomembrane Na^+^/H^+^ antiporter gene from *Chrysanthemum crissum*; *CmSOS1*, cytomembrane Na^+^/H^+^ antiporter gene from *Chrysanthemum morifolium*; *CrcSOS1*, cytomembrane Na^+^/H^+^ antiporter gene from *Crossostephium chinense*; *AjSOS1*, cytomembrane Na^+^/H^+^ antiporter gene from *Artemisia japonica*; *LfSOS1*, cytomembrane Na^+^/H^+^ antiporter gene from *Leptochloa fusca*; *LfNHX1*, tonoplast Na^+^/H^+^ antiporter gene from *Leptochloa fusca*; *IlNHX*, tonoplast Na^+^/H^+^ antiporter gene from *Iris lacteal*; *HtNHX1 and HtNHX2*, tonoplast Na^+^/H^+^ antiporter gene from *Helianthus tuberosus*; *AlHKT2:1*, high-affinity K^+^ transporter gene from *Aeluropus lagopoides*; *PsPSK1*, a SKP1-like gene homologue from *Paeonia suffruticosa*; *DgNAC1*, *NAC* transcription factor gene from *Dendronthema grandiform*; *DgWRKY4*, *WRKY* transcription factor gene of from *Dendronthema grandiform*; *CpbHLH13*, *bHLH* transcription factor gene from *Chimonanthus praecox*; and *CpMYC2*, *bHLH* transcription factor gene from *Chimonanthus praecox*.

Further work based on the genome sequencing of ornamental plants (Wani et al., 2020) will lay the foundation for subsequent gene editing to obtain highly salt-tolerant ornamental flowers for greening saline–alkaline areas. Transcriptome analysis of ornamental plants will also reveal previously unknown genes related to flower color or quality, and genes related to the response to a saline environment (Guo et al., 2019b). Current methods of obtaining novel varieties are shown in Figure 2.

## Conclusion and Perspectives

**Figure 3:**
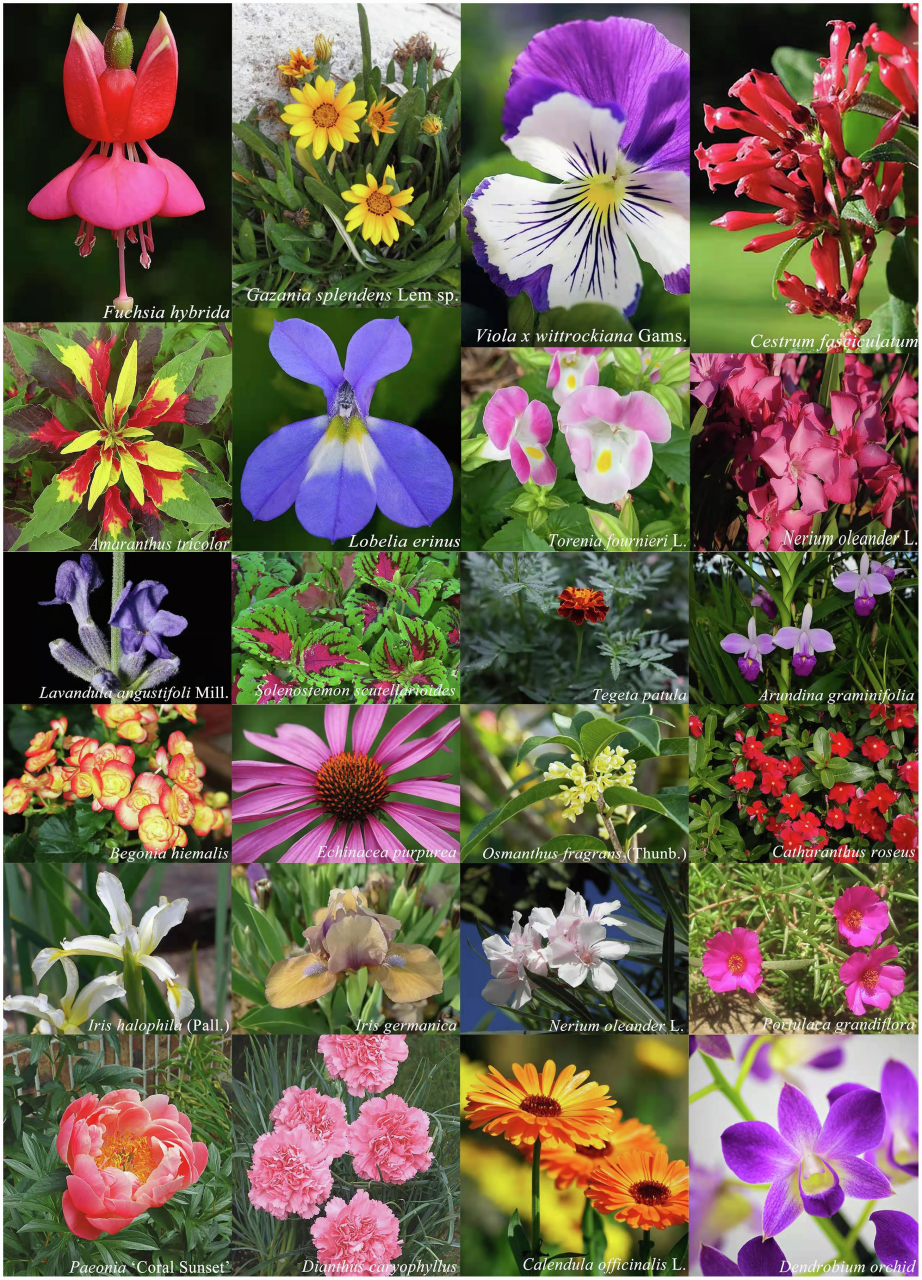
Photos exhibition of some ornamental plants with beautiful flowers.

Saline soils are widely distributed around the world, and these are considered to be a valuable land resource for development. Protection by vegetation and “greening” is the most effective and feasible biological methods for improving saline lands. Photos of some ornamental plants with beautiful flowers are shown in Figure 3. Planting salt-tolerant ornamental flowering crops is a feasible and sustainable greening strategy. Achieving this goal relies on selecting and breeding ornamental flowering crop varieties with both high salt stress tolerance and high value.

Like most plants in a salinized environment, the responses or adaptive strategies of ornamental flowering crops to salt aim to mitigate the effects of osmotic, ionic, and oxidative stresses, as well as nutritional imbalances induced by salinity. Under saline conditions, ornamental plants accumulate osmolytes like proline, redistribute ions, enhance their ROS scavenging ability, reduce the salt ion uptake by roots, and secrete salt ions from leaves.

With the help of genetic engineering approaches such as genetic modification and gene editing, future studies should focus on understanding the salt tolerance mechanisms of ornamental flowering crops at the molecular level, and on providing the basis for breeding ornamental flowering crops with high salt tolerance, more flower colors, and greater economic value. Other strategies include selection and acceptance of model ornamental flowering plants with high salt tolerance; genome sequencing of model plants; and establishment of highly efficient transgenic systems for model plants. These strategies would form the basis for deciphering the salt-tolerant mechanisms of ornamental plants at the molecular levels; for example, they could provide insight into the role of the apoplastic barrier in ornamental plant roots for saline tolerance. Novel ornamental plant cultivars with high salt tolerance and expanded usages will be obtained using modern biological techniques.

## Author Contributions

JG and BW conceived and designed this study and revised the manuscript. JG, CS, and YifanZ wrote the manuscript. XW and HT collected the literatures. GH and YiZ proposed related theories. All authors contributed to the article and approved the submitted version.

## Funding

The study was supported by the financial funding of the National Natural Science Foundation of China (31770288 and 31800304), the Natural Science Research Foundation of Shandong Province (ZR2017MC003), and the Higher Educational Science and Technology Program of Shandong Province (J17KA136).

## Conflict of Interest

The authors declare that the research was conducted in the absence of any commercial or financial relationships that could be construed as a potential conflict of interest.

## Publisher’s Note

All claims expressed in this article are solely those of the authors and do not necessarily represent those of their affiliated organizations, or those of the publisher, the editors and the reviewers. Any product that may be evaluated in this article, or claim that may be made by its manufacturer, is not guaranteed or endorsed by the publisher.

## Supplementary Material

Supplementary Figure S1 Summarized structural adaptation to salinity of halophytic ornamental plants. Notes: (i) pumping Na^+^ back to the environment; (ii) depositing large amounts of salt ions in vacuoles of succulent tissues; (iii) secreting salt ions through salt bladder; (iv) secreting salt ions through salt glands. MC, mesophyll cell; EC, epidermal cell; OC, outer cup cell; IC, inner cup cell; AC, accessory cell; and SC, secretory cell.

## References

[ref1] Abdullakasim S. Kongpaisan P. Thongjang P. Saradhuldhat P. (2018). Physiological responses of potted *Dendrobium orchid* to salinity stress. Hortic. Environ. Biotechnol. 59, 491–498. doi: 10.1007/s13580-018-0057-4

[ref2] Aboutalebi Jahromi A. Hosseini Farahi M. (2016). Seed germination, vegetative growth and concentration of some elements in French marigold (*Tageta patula*) as influenced by salinity and ammonium nitrate. Int. J. Hortic. Sci. Technol. 3, 199–209. doi: 10.22059/ijhst.2017.212510.145

[ref3] Acosta-Motos J.-R. Diaz-Vivancos P. Álvarez S. Fernández-García N. Sanchez-Blanco M. J. Hernández J. A. (2015). Physiological and biochemical mechanisms of the ornamental *Eugenia myrtifolia* L. plants for coping with NaCl stress and recovery. Planta 242, 829–846. doi: 10.1007/s00425-015-2315-3, PMID: 25976265

[ref4] Acosta-Motos J. R. Hernández J. A. Álvarez S. Barba-Espín G. Sánchez-Blanco M. J. (2017). The long-term resistance mechanisms, critical irrigation threshold and relief capacity shown by *Eugenia myrtifolia* plants in response to saline reclaimed water. Plant Physiol. Biochem. 111, 244–256. doi: 10.1016/j.plaphy.2016.12.003, PMID: 27951494

[ref5] Adamipour N. Khosh-Khui M. Salehi H. Rho H. (2019). Effect of vermicompost on morphological and physiological performances of pot marigold (*Calendula officinalis* L.) under salinity conditions. Adv. Hortic. Sci. 33, 345–358. doi: 10.13128/ahs-23714

[ref6] Ahmad J. Bashir H. Bagheri R. Baig A. Al-Huqail A. Ibrahim M. M.. (2017). Drought and salinity induced changes in ecophysiology and proteomic profile of *Parthenium hysterophorus*. PLoS One 12:e0185118. doi: 10.1371/journal.pone.0185118, PMID: 28953916 PMC5617186

[ref7] Ahmad S. Lu C. Wu J. Wei Y. Gao J. Jin J.. (2021). Transcriptional cascade in the regulation of flowering in the bamboo orchid *Arundina graminifolia*. Biomol. Ther. 11:771. doi: 10.3390/biom11060771, PMID: 34063940 PMC8224086

[ref8] Ali A. Iqbal N. Ali F. Afzal B. (2012). Alternanthera bettzickiana (regel) G. Nicholson, a potential halophytic ornamental plant: growth and physiological adaptations. Flora: Morphol. Distrib. Funct. Ecol. Plants 207, 318–321. doi: 10.1016/j.flora.2011.12.002

[ref9] Ali A. Raddatz N. Aman R. Kim S. Park H. C. Jan M.. (2016). A single amino-acid substitution in the sodium transporter HKT1 associated with plant salt tolerance. Plant Physiol. 171, 2112–2126. doi: 10.1104/pp.16.00569, PMID: 27208305 PMC4936583

[ref10] Álvarez S. Sanchez-Blanco M. (2014). Long-term effect of salinity on plant quality, water relations, photosynthetic parameters and ion distribution in *Callistemon citrinus*. Plant Biol. 16, 757–764. doi: 10.1111/plb.12106, PMID: 24118672

[ref11] An J. Song A. Guan Z. Jiang J. Chen F. Lou W.. (2014). The over-expression of *Chrysanthemum crassum CcSOS1* improves the salinity tolerance of chrysanthemum. Mol. Biol. Rep. 41, 4155–4162. doi: 10.1007/s11033-014-3287-2, PMID: 24566689

[ref12] Andersen T. G. Barberon M. Geldner N. (2015). Suberization—the second life of an endodermal cell. Curr. Opin. Plant Biol. 28, 9–15. doi: 10.1016/j.pbi.2015.08.004, PMID: 26343015

[ref13] Anne S. Lim J. H. (2020). Mutation breeding using gamma irradiation in the development of ornamental plants: a review. Flower Res. J. 28, 102–115. doi: 10.11623/frj.2020.28.3.01

[ref14] Ayad J. Othman Y. Al Antary T. (2019). Irrigation water salinity and potassium enrichment influenced growth and flower quality of Asiatic lily. Fresenius Environ. Bull. 28, 8900–8905.

[ref15] Azizi F. Farsaraei S. Moghaddam M. (2021). Application of exogenous ascorbic acid modifies growth and pigment content of *Calendula officinalis* L. flower heads of plants exposed to NaCl stress. J. Soil Sci. Plant Nutr. 21, 1–12. doi: 10.1007/s42729-021-00567-0

[ref16] Bai Y. Kissoudis C. Yan Z. Visser R. G. van der Linden G. (2018). Plant behaviour under combined stress: tomato responses to combined salinity and pathogen stress. Plant J. 93, 781–793. doi: 10.1111/tpj.13800, PMID: 29237240

[ref17] Blumwald E. (2000). Sodium transport and salt tolerance in plants. Curr. Opin. Cell Biol. 12, 431–434. doi: 10.1016/S0955-0674(00)00112-5 10873827

[ref18] Borsai O. Hassan M. A. Negrușier C. Raigón M. D. Boscaiu M. Sestraș R. E.. (2020). Responses to salt stress in *Portulaca*: insight into its tolerance mechanisms. Plan. Theory 9:1660. doi: 10.3390/plants9121660, PMID: 33260911 PMC7760961

[ref19] Boutigny A.-L. Dohin N. Pornin D. Rolland M. (2020). Overview and detectability of the genetic modifications in ornamental plants. Hortic. Res. 7, 1–12. doi: 10.1038/s41438-019-0232-5 32025314 PMC6994484

[ref20] Bowler C. Montagu M. V. Inze D. (1992). Superoxide dismutase and stress tolerance. Annu. Rev. Plant Physiol. Plant Mol. Biol. 43, 83–116. doi: 10.1146/annurev.pp.43.060192.000503

[ref21] Breś W. Bandurska H. Kupska A. Niedziela J. Frąszczak B. (2016). Responses of pelargonium (*Pelargonium× hortorum* LH bailey) to long-term salinity stress induced by treatment with different NaCl doses. Acta Physiol. Plant. 38, 1–11. doi: 10.1007/s11738-015-2048-8

[ref22] Cassaniti C. Leonardi C. Flowers T. J. (2009). The effects of sodium chloride on ornamental shrubs. Sci. Hortic. 122, 586–593. doi: 10.1016/j.scienta.2009.06.032

[ref23] Caverzan A. Casassola A. Brammer S. P. (2016). “Reactive oxygen species and antioxidant enzymes involved in plant tolerance to stress,” in Abiotic and Biotic Stress in Plants-Recent Advances and Future Perspectives. eds. Shanker A. K. Shanker C. (London: InTech), 463–480.

[ref24] Chen H. Jiang J.-G. (2010). Osmotic adjustment and plant adaptation to environmental changes related to drought and salinity. Environ. Rev. 18, 309–319. doi: 10.1139/A10-014

[ref25] Chen M. Song J. Wang B.-S. (2010). NaCl increases the activity of the plasma membrane H^+^-ATPase in C_3_ halophyte *Suaeda salsa* callus. Acta Physiol. Plant. 32, 27–36. doi: 10.1007/s11738-009-0371-7

[ref26] Chinnusamy V. Jagendorf A. Zhu J. K. (2005). Understanding and improving salt tolerance in plants. Crop Sci. 45, 437–448. doi: 10.2135/cropsci2005.0437

[ref27] Chrysargyris A. Tzionis A. Xylia P. Tzortzakis N. (2018). Effects of salinity on tagetes growth, physiology, and shelf life of edible flowers stored in passive modified atmosphere packaging or treated with ethanol. Front. Plant Sci. 9:1765. doi: 10.3389/fpls.2018.01765, PMID: 30619383 PMC6296340

[ref28] Cirillo C. Rouphael Y. Caputo R. Raimondi G. Sifola M. De Pascale S. (2016). Effects of high salinity and the exogenous application of an osmolyte on growth, photosynthesis, and mineral composition in two ornamental shrubs. J. Hortic. Sci. Biotechnol. 91, 14–22. doi: 10.1080/14620316.2015.1110988

[ref29] Dave A. Sanadhya P. Joshi P. S. Agarwal P. Agarwal P. K. (2021). Molecular cloning and characterization of high-affinity potassium transporter (*AlHKT2;1*) gene promoter from halophyte *Aeluropus lagopoides*. Int. J. Biol. Macromol. 181, 1254–1264. doi: 10.1016/j.ijbiomac.2021.05.038, PMID: 33989688

[ref30] Davenport R. J. Muñoz-Mayor A. Jha D. Essah P. A. Rus A. Tester M. (2007). The Na^+^ transporter AtHKT1;1 controls retrieval of Na^+^ from the xylem in Arabidopsis. Plant Cell Environ. 30, 497–507. doi: 10.1111/j.1365-3040.2007.01637.x, PMID: 17324235

[ref31] Ding G. Yang Q. Ruan X. Si T. Yuan B. Zheng W.. (2022). Proteomics analysis of the effects for different salt ions in leaves of true halophyte *Sesuvium portulacastrum*. Plant Physiol. Biochem. 170, 234–248. doi: 10.1016/j.plaphy.2021.12.009, PMID: 34920320

[ref32] Duarte B. Cabrita M. Gameiro C. Matos A. Godinho R. Marques J. C.. (2017). Disentangling the photochemical salinity tolerance in *Aster tripolium* L.: connecting biophysical traits with changes in fatty acid composition. Plant Biol. 19, 239–248. doi: 10.1111/plb.12517, PMID: 27748562

[ref33] El-Shawa G. M. Rashwan E. M. Abdelaal K. A. (2020). Mitigating salt stress effects by exogenous pplication of proline and yeast extract on morpho-physiological, biochemical and anatomical characters of calendula plants. Sci. J. Flower. Ornam. Plants 7, 461–482. doi: 10.21608/sjfop.2020.135166

[ref34] Escalona A. Salas M. D. C. Coutinho C. D. S. Guzmán M. (2013). How does salinity affect mineral ion relations and growth of *Lobelia erinus* for use in urban landscaping. J. Food Agric. Environ. 11, 854–858.

[ref35] Feng Z. Deng Y. Fan H. Sun Q. Sui N. Wang B. (2014a). Effects of NaCl stress on the growth and photosynthetic characteristics of *Ulmus pumila* L. seedlings in sand culture. Photosynthetica 52, 313–320. doi: 10.1007/s11099-014-0032-y

[ref36] Feng Z.-T. Deng Y.-Q. Zhang S.-C. Liang X. Yuan F. Hao J.-L.. (2015). K^+^ accumulation in the cytoplasm and nucleus of the salt gland cells of *Limonium bicolor* accompanies increased rates of salt secretion under NaCl treatment using NanoSIMS. Plant Sci. 238, 286–296. doi: 10.1016/j.plantsci.2015.06.021, PMID: 26259195

[ref37] Feng Z. Sun Q. Deng Y. Sun S. Zhang J. Wang B. (2014b). Study on pathway and characteristics of ion secretion of salt glands of *Limonium bicolor*. Acta Physiol. Plant. 36, 2729–2741. doi: 10.1007/s11738-014-1644-3

[ref38] Gao J. Sun J. Cao P. Ren L. Liu C. Chen S.. (2016). Variation in tissue Na^+^ content and the activity of *SOS1* genes among two species and two related genera of chrysanthemum. BMC Plant Biol. 16:98. doi: 10.1186/s12870-016-0781-9, PMID: 27098270 PMC4839091

[ref39] García-Caparrós P. Lao M. T. (2018). The effects of salt stress on ornamental plants and integrative cultivation practices. Sci. Hortic. 240, 430–439. doi: 10.1016/j.scienta.2018.06.022

[ref40] García-Caparrós P. Llanderal A. Pestana M. Correia P. J. Lao M. T. (2016). Tolerance mechanisms of three potted ornamental plants grown under moderate salinity. Sci. Hortic. 201, 84–91. doi: 10.1016/j.scienta.2016.01.031

[ref41] Geng X. Zhang Y. Wang L. Yang X. (2019). Pretreatment with high-dose gamma irradiation on seeds enhances the tolerance of sweet osmanthus seedlings to salinity stress. Forests 10:406. doi: 10.3390/f10050406

[ref42] Gómez Bellot M. J. Carmassi G. Bartalucci M. Sánchez-Blanco M. J. Pardossi A. (2018). Growth, evapotranspiration and mineral content of gerbera under combined salinity and excess boron. J. Hortic. Res. 26, 61–69. doi: 10.2478/johr-2018-0017

[ref43] González-Orenga S. Grigore M.-N. Boscaiu M. Vicente O. (2021). Constitutive and induced salt tolerance mechanisms and potential uses of *Limonium* mill. Species. Agronomy 11:413. doi: 10.3390/agronomy11030413

[ref44] Guo J. Dong X. Han G. Wang B. (2019a). Salt-enhanced reproductive development of *Suaeda salsa* L. coincided with ion transporter gene upregulation in flowers and increased pollen K^+^ content. Front. Plant Sci. 10:333. doi: 10.3389/fpls.2019.00333, PMID: 30984214 PMC6449877

[ref45] Guo J. Li Y. Han G. Song J. Wang B. (2018). NaCl markedly improved the reproductive capacity of the euhalophyte *Suaeda salsa*. Funct. Plant Biol. 45, 350–361. doi: 10.1071/FP17181, PMID: 32290958

[ref46] Guo J. Suo S. Wang B.-S. (2015). Sodium chloride improves seed vigour of the euhalophyte *Suaeda salsa*. Seed Sci. Res. 25, 335–344. doi: 10.1017/S0960258515000239

[ref47] Guo Q. Tian X. Mao P. Meng L. (2020). Overexpression of Iris lactea tonoplast Na^+^/H^+^ antiporter gene *IlNHX* confers improved salt tolerance in tobacco. Biol. Plant. 64, 50–57. doi: 10.32615/bp.2019.126

[ref48] Guo L. Wang Y. da Silva J. A. T. Fan Y. Yu X. (2019b). Transcriptome and chemical analysis reveal putative genes involved in flower color change in *Paeonia* ‘coral sunset’. Plant Physiol. Biochem. 138, 130–139. doi: 10.1016/j.plaphy.2019.02.025, PMID: 30870763

[ref49] Han N. Lan W. He X. Shao Q. Wang B. Zhao X. (2012). Expression of a *Suaeda salsa* vacuolar H^+^/Ca^2+^ transporter gene in Arabidopsis contributes to physiological changes in salinity. Plant Mol. Biol. Report. 30, 470–477. doi: 10.1007/s11105-011-0353-y

[ref50] Hao Q. Ren H. Zhu J. Wang L. Huang S. Liu Z.. (2017). Overexpression of *PSK1, a SKP1-like* gene homologue, from *Paeonia suffruticosa*, confers salinity tolerance in Arabidopsis. Plant Cell Rep. 36, 151–162. doi: 10.1007/s00299-016-2066-z, PMID: 27787596

[ref51] Hasegawa P. M. (2013). Sodium (Na^+^) homeostasis and salt tolerance of plants. Environ. Exp. Bot. 92, 19–31. doi: 10.1016/j.envexpbot.2013.03.001

[ref52] Hose E. Clarkson D. T. Steudle E. Schreiber L. Hartung W. (2001). The exodermis: a variable apoplastic barrier. J. Exp. Bot. 52, 2245–2264. doi: 10.1093/jexbot/52.365.2245, PMID: 11709575

[ref53] Hsouna A. B. Ghneim-Herrera T. Romdhane W. B. Dabbous A. Saad R. B. Brini F.. (2020). Early effects of salt stress on the physiological and oxidative status of the halophyte *Lobularia maritima*. Funct. Plant Biol. 47, 912–924. doi: 10.1071/FP19303, PMID: 32611480

[ref54] Jaleel C. A. Gopi R. Manivannan P. Gomathinayagam M. Murali P. Panneerselvam R. (2008). Soil applied propiconazole alleviates the impact of salinity on *Catharanthus roseus* by improving antioxidant status. Pestic. Biochem. Physiol. 90, 135–139. doi: 10.1016/j.pestbp.2007.11.003

[ref55] Jiang C. Zheng Q. Liu Z. Liu L. Zhao G. Long X.. (2011). Seawater-irrigation effects on growth, ion concentration, and photosynthesis of transgenic poplar overexpressing the Na^+^/H^+^ antiporter AtNHX1. J. Plant Nutr. Soil Sci. 174, 301–310. doi: 10.1002/jpln.201000033

[ref56] Koksal N. Alkan-Torun A. Kulahlioglu I. Ertargin E. Karalar E. (2016). Ion uptake of marigold under saline growth conditions. Springerplus 5, 1–12. doi: 10.1186/s40064-016-1815-3 26933637 PMC4761351

[ref57] Kozi A. (1999). The water-water cycle in chloroplasts: scavenging of active oxygens and dissipation of excess photons. Annu. Rev. Plant Physiol. Plant Mol. Biol. 50, 601–639. doi: 10.1146/annurev.arplant.50.1.601 15012221

[ref001] Krishnamurthy P. Ranathunge K. Nayak S. Schreiber L. Mathew M. (2011). Root apoplastic barriers block Na+ transport to shoots in rice (*Oryza sativa* L.). J. Exp. Bot. 62, 4215–4228. doi: 10.1093/jxb/err135 21558150 PMC3153681

[ref58] Krishnamurthy P. Vishal B. Ho W. J. Lok F. C. J. Lee F. S. M. Kumar P. P. (2020). Regulation of a cytochrome P450 gene *CYP94B1* by WRKY33 transcription factor controls apoplastic barrier formation in roots to confer salt tolerance. Plant Physiol. 184, 2199–2215. doi: 10.1104/pp.20.01054, PMID: 32928900 PMC7723105

[ref59] Kumar D. Al Hassan M. Naranjo M. A. Agrawal V. Boscaiu M. Vicente O. (2017). Effects of salinity and drought on growth, ionic relations, compatible solutes and activation of antioxidant systems in oleander (*Nerium oleander* L.). PLoS One 12:e0185017. doi: 10.1371/journal.pone.0185017, PMID: 28922384 PMC5602669

[ref60] Kwon O. K. Mekapogu M. Kim K. S. (2019). Effect of salinity stress on photosynthesis and related physiological responses in carnation (*Dianthus caryophyllus*). Hortic. Environ. Biotechnol. 60, 831–839. doi: 10.1007/s13580-019-00189-7

[ref61] Leng B. Yuan F. Dong X. Wang J. Wang B. (2018). Distribution pattern and salt excretion rate of salt glands in two recretohalophyte species of *Limonium* (Plumbaginaceae). S. Afr. J. Bot. 115, 74–80. doi: 10.1016/j.sajb.2018.01.002

[ref62] Li K. Pang C.-H. Ding F. Sui N. Feng Z.-T. Wang B.-S. (2012). Overexpression of *Suaeda salsa* stroma ascorbate peroxidase in Arabidopsis chloroplasts enhances salt tolerance of plants. S. Afr. J. Bot. 78, 235–245. doi: 10.1016/j.sajb.2011.09.006

[ref63] Liang M. Lin M. Lin Z. Zhao L. Zhao G. Li Q.. (2015). Identification, functional characterization, and expression pattern of a NaCl-inducible vacuolar Na^+^/H^+^ antiporter in chicory (*Cichorium intybus* L.). Plant Growth Regul. 75, 605–614. doi: 10.1007/s10725-014-9963-3

[ref64] Liu Q. Tang J. Wang W. Zhang Y. Yuan H. Huang S. (2018). Transcriptome analysis reveals complex response of the medicinal/ornamental halophyte Iris halophila pall. To high environmental salinity. Ecotox. Environ. Safe 165, 250–260. doi: 10.1016/j.ecoenv.2018.09.003, PMID: 30199796

[ref65] Lu C. Feng Z. Yuan F. Han G. Guo J. Chen M.. (2020). The SNARE protein LbSYP61 participates in salt secretion in *Limonium bicolor*. Environ. Exp. Bot. 176:104076. doi: 10.1016/j.envexpbot.2020.104076

[ref66] Luo X. Wu J. Li Y. Nan Z. Guo X. Wang Y.. (2013). Synergistic effects of *GhSOD1* and *GhCAT1* overexpression in cotton chloroplasts on enhancing tolerance to methyl viologen and salt stresses. PLoS One 8:e54002. doi: 10.1371/journal.pone.0054002, PMID: 23335985 PMC3545958

[ref67] Meyer C. J. Seago J. L. Jr. Peterson C. A. (2009). Environmental effects on the maturation of the endodermis and multiseriate exodermis of *Iris germanica* roots. Ann. Bot. 103, 687–702. doi: 10.1093/aob/mcn255, PMID: 19151041 PMC2707867

[ref68] Mi P. Yuan F. Guo J. Han G. Wang B. (2021). Salt glands play a pivotal role in the salt resistance of four recretohalophyte *Limonium* mill. Species. Plant Biol. 23, 1063–1073. doi: 10.1111/plb.13284, PMID: 33969585

[ref69] Mittler R. (2002). Oxidative stress, antioxidants and stress tolerance. Trends Plant Sci. 7, 405–410. doi: 10.1016/S1360-1385(02)02312-9 12234732

[ref70] Mohammadi F. Kavousi H. R. Mansouri M. (2019). Effects of salt stress on physio-biochemical characters and gene expressions in halophyte grass *Leptochloa fusca* (L.) Kunth. Acta Physiol. Plant. 41, 1–10. doi: 10.1007/s11738-019-2935-5

[ref71] Munns R. Tester M. (2008). Mechanisms of salinity tolerance. Annu. Rev. Plant Biol. 59, 651–681. doi: 10.1146/annurev.arplant.59.032607.092911, PMID: 18444910

[ref72] Nakhaie A. Habibi G. Vaziri A. (2020). Exogenous proline enhances salt tolerance in acclimated *Aloe vera* by modulating photosystem II efficiency and antioxidant defense. S. Afr. J. Bot. 06:005. doi: 10.1016/j.sajb.2020.06.005

[ref73] Nikalje G. C. Variyar P. S. Joshi M. V. Nikam T. D. Suprasanna P. (2018). Temporal and spatial changes in ion homeostasis, antioxidant defense and accumulation of flavonoids and glycolipid in a halophyte *Sesuvium portulacastrum* (L.) L. PLoS One 13:e0193394. doi: 10.1371/journal.pone.0193394, PMID: 29641593 PMC5894978

[ref74] Nishihara M. Higuchi A. Watanabe A. Tasaki K. (2018). Application of the CRISPR/Cas9 system for modification of flower color in *Torenia fournieri*. BMC Plant Biol. 18:331. doi: 10.1186/s12870-018-1539-3, PMID: 30518324 PMC6280492

[ref75] Oh D.-H. Leidi E. Zhang Q. Hwang S.-M. Li Y. Quintero F. J.. (2009). Loss of halophytism by interference with SOS1 expression. Plant Physiol. 151, 210–222. doi: 10.1104/pp.109.137802, PMID: 19571313 PMC2735974

[ref76] Pang C. H. Li K. Wang B. (2011). Overexpression of SsCHLAPXs confers protection against oxidative stress induced by high light in transgenic *Arabidopsis thaliana*. Physiol. Plant. 143, 355–366. doi: 10.1111/j.1399-3054.2011.01515.x, PMID: 21895668

[ref77] Pušić M. G. Mladenović E. M. Čukanović J. D. Lakićević M. D. Pavlović L. M. (2019). Influence of salinity on the growth and development of pansies (*Viola x wittrockiana Gams*.). Zbornik Matice Srpske za Prirodne Nauke 2019, 57–66. doi: 10.2298/ZMSPN1937057P

[ref78] Qi X. Tam N. F.-Y. Li W. C. Ye Z. (2020). The role of root apoplastic barriers in cadmium translocation and accumulation in cultivars of rice (*Oryza sativa* L.) with different cd-accumulating characteristics. Environ. Pollut. 264:114736. doi: 10.1016/j.envpol.2020.114736, PMID: 32417578

[ref79] Qiu N. Chen M. Guo J. Bao H. Ma X. Wang B. (2007). Coordinate up-regulation of V-H^+^-ATPase and vacuolar Na^+^/H^+^ antiporter as a response to NaCl treatment in a C_3_ halophyte *Suaeda salsa*. Plant Sci. 172, 1218–1225. doi: 10.1016/j.plantsci.2007.02.013

[ref80] Rahi T. Singh B. (2011). Salinity tolerance in *Chrysanthemum morifolium*. J. Appl. Hortic. 13, 30–36. doi: 10.37855/jah.2011.v13i01.07

[ref81] Reddy A. R. Chaitanya K. V. Vivekanandan M. (2004). Drought-induced responses of photosynthesis and antioxidant metabolism in higher plants. J. Plant Physiol. 161, 1189–1202. doi: 10.1016/j.jplph.2004.01.013, PMID: 15602811

[ref82] Roychoudhury A. Banerjee A. (2016). Endogenous glycine betaine accumulation mediates abiotic stress tolerance in plants. Trop. Plant Res. 3, 105–111.

[ref83] Rus A. Yokoi S. Sharkhuu A. Reddy M. Lee B.-H. Matsumoto T. K.. (2001). AtHKT1 is a salt tolerance determinant that controls Na^+^ entry into plant roots. Proc. Natl. Acad. Sci. 98, 14150–14155. doi: 10.1073/pnas.241501798, PMID: 11698666 PMC61183

[ref84] Sabra A. Daayf F. Renault S. (2012). Differential physiological and biochemical responses of three *Echinacea* species to salinity stress. Sci. Hortic. 135, 23–31. doi: 10.1016/j.scienta.2011.11.024

[ref85] Sarker U. Oba S. (2020). The response of salinity stress-induced *A. tricolor* to growth, anatomy, physiology, non-enzymatic and enzymatic antioxidants. Front. Plant Sci. 11:559876. doi: 10.3389/fpls.2020.559876, PMID: 33178233 PMC7596248

[ref86] Shabala S. Wu H. Bose J. (2015). Salt stress sensing and early signalling events in plant roots: current knowledge and hypothesis. Plant Sci. 241, 109–119. doi: 10.1016/j.plantsci.2015.10.003, PMID: 26706063

[ref87] Shao Q. Han N. Ding T. Zhou F. Wang B. (2014). *SsHKT1; 1* is a potassium transporter of the C_3_ halophyte *Suaeda salsa* that is involved in salt tolerance. Funct. Plant Biol. 41, 790–802. doi: 10.1071/FP13265, PMID: 32481033

[ref88] Shi H. Ishitani M. Kim C. Zhu J.-K. (2000). The Arabidopsis thaliana salt tolerance gene *SOS1* encodes a putative Na^+^/H^+^ antiporter. Proc. Natl. Acad. Sci. 97, 6896–6901. doi: 10.1073/pnas.120170197, PMID: 10823923 PMC18772

[ref89] Song J. (2009). Root morphology is related to the phenotypic variation in waterlogging tolerance of two populations of *Suaeda salsa* under salinity. Plant Soil 324, 231–240. doi: 10.1007/s11104-009-9949-5

[ref90] Song A. Lu J. Jiang J. Chen S. Guan Z. Fang W.. (2012). Isolation and characterisation of *Chrysanthemum crassum* SOS1, encoding a putative plasma membrane Na^+^/H^+^ antiporter. Plant Biol. 14, 706–713. doi: 10.1111/j.1438-8677.2011.00560.x, PMID: 22404736

[ref91] Song J. Shi W. Liu R. Xu Y. Sui N. Zhou J.. (2017). The role of the seed coat in adaptation of dimorphic seeds of the euhalophyte *Suaeda salsa* to salinity. Plant Spec. Biol. 32, 107–114. doi: 10.1111/1442-1984.12132

[ref92] Song J. Wang B. (2015). Using euhalophytes to understand salt tolerance and to develop saline agriculture: *Suaeda salsa* as a promising model. Ann. Bot. 115, 541–553. doi: 10.1093/aob/mcu194, PMID: 25288631 PMC4332605

[ref93] Song J. Zhou J. Zhao W. Xu H. Wang F. Xu Y.. (2016). Effects of salinity and nitrate on production and germination of dimorphic seeds applied both through the mother plant and exogenously during germination in *Suaeda salsa*. Plant Spec. Biol. 31, 19–28. doi: 10.1111/1442-1984.12071

[ref94] Soroori S. Danaee E. Hemmati K. Moghadam A. L. Garmsar I. (2021). Effect of foliar application of proline on morphological and physiological traits of *Calendula officinalis* L. under drought stress. J. Ornam. Plants 11, 13–30.

[ref95] Souid A. Gabriele M. Longo V. Pucci L. Bellani L. Smaoui A.. (2016). Salt tolerance of the halophyte *Limonium delicatulum* is more associated with antioxidant enzyme activities than phenolic compounds. Funct. Plant Biol. 43, 607–619. doi: 10.1071/FP15284 32480490

[ref96] Szekely-Varga Z. Gonzalez-Orenga S. Cantor M. Boscaiu M. Vicente O. (2020). Antioxidant responses to drought and salinity in *Lavandula angustifolia* mill. Not. Bot. Horti Agrobot. 48, 1980–1992. doi: 10.15835/nbha48412150 PMC7284986 32429357

[ref97] Tang X. Mu X. Shao H. Wang H. Brestic M. (2015). Global plant-responding mechanisms to salt stress: physiological and molecular levels and implications in biotechnology. Crit. Rev. Biotechnol. 35, 425–437. doi: 10.3109/07388551.2014.889080, PMID: 24738851

[ref98] Ulczycka-Walorska M. KrzymiŃSka A. Bandurska H. Bocianowski J. (2020). Response of *Hyacinthus orientalis* L. to salinity caused by increased concentrations of sodium chloride in the soil. Not. Bot. Horti Agrobot. 48, 398–405. doi: 10.15835/nbha48111748

[ref99] Van Zelm E. Zhang Y. Testerink C. (2020). Salt tolerance mechanisms of plants. Annu. Rev. Plant Biol. 71, 403–433. doi: 10.1146/annurev-arplant-050718-100005, PMID: 32167791

[ref100] Vanlalruati A. Kumar G. Tiwari A. (2019). Effect of saline stress on growth and biochemical indices of chrysanthemum (*Chrysanthemum morifolium*) germplasm. Indian J. Agric. Sci. 89, 41–45.

[ref101] Veatch-Blohm M. E. Sawch D. Elia N. Pinciotti D. (2014). Salinity tolerance of three commonly planted *narcissus* cultivars. HortScience 49, 1158–1164. doi: 10.21273/HORTSCI.49.9.1158

[ref102] Villarino G. H. Mattson N. S. (2011). Assessing tolerance to sodium chloride salinity in fourteen floriculture species. HortTechnology 21, 539–545. doi: 10.21273/HORTTECH.21.5.539

[ref103] Wang Q. Guan C. Wang S.-M. (2014). Coordination of AtHKT1; 1 and AtSOS1 facilitates Na+ and K+ homeostasis in *Arabidopsis thaliana* under salt stress. J. Plant Biol. 57, 282–290. doi: 10.1007/s12374-014-0222-y

[ref104] Wang Q. Guan C. Wang P. Ma Q. Bao A.-K. Zhang J.-L.. (2019a). The effect of AtHKT1;1 or AtSOS1 mutation on the expressions of Na^+^ or K^+^ transporter genes and ion homeostasis in *Arabidopsis thaliana* under salt stress. Int. J. Mol. Sci. 20:1085. doi: 10.3390/ijms20051085, PMID: 30832374 PMC6429264

[ref105] Wang H. Li T. Li W. Wang W. Zhao H. (2021). Identification and analysis of *chrysanthemum nankingense* NAC transcription factors and an expression analysis of OsNAC7 subfamily members. PeerJ 9:e11505. doi: 10.7717/peerj.11505, PMID: 34123596 PMC8164415

[ref106] Wang Y. Sun Y. Niu G. Deng C. Wang Y. Gardea-Torresdey J. (2019c). Growth, gas exchange, and mineral nutrients of ornamental grasses irrigated with saline water. HortScience 54, 1840–1846. doi: 10.21273/HORTSCI13953-19

[ref107] Wang K. Wu Y.-H. Tian X.-Q. Bai Z.-Y. Liang Q.-Y. Liu Q.-L.. (2017a). Overexpression of *DgWRKY4* enhances salt tolerance in chrysanthemum seedlings. Front. Plant Sci. 8:1592. doi: 10.3389/fpls.2017.01592, PMID: 28959270 PMC5604078

[ref108] Wang W. Xu Y. Chen T. Xing L. Xu K. Ji D.. (2019b). Regulatory mechanisms underlying the maintenance of homeostasis in *Pyropia haitanensis* under hypersaline stress conditions. Sci. Total Environ. 662, 168–179. doi: 10.1016/j.scitotenv.2019.01.214, PMID: 30690352

[ref109] Wang F. Xu Y.-G. Wang S. Shi W. Liu R. Feng G.. (2015). Salinity affects production and salt tolerance of dimorphic seeds of *Suaeda salsa*. Plant Physiol. Biochem. 95, 41–48. doi: 10.1016/j.plaphy.2015.07.005, PMID: 26184090

[ref110] Wang F. Yin C. Song Y. Li Q. Tian C. Song J. (2018). Reproductive allocation and fruit-set pattern in the euhalophyte Suaeda salsa in controlled and field conditions. Plant Biosyst. 152, 749–758. doi: 10.1080/11263504.2017.1330776

[ref111] Wang B. Zhai H. He S. Zhang H. Ren Z. Zhang D.. (2016). A vacuolar Na^+^/H^+^ antiporter gene, *IbNHX2*, enhances salt and drought tolerance in transgenic sweetpotato. Sci. Hortic. 201, 153–166. doi: 10.1016/j.scienta.2016.01.027

[ref112] Wang K. Zhong M. Wu Y. Bai Z. Liang Q. Liu Q.. (2017b). Overexpression of a chrysanthemum transcription factor gene *DgNAC1* improves the salinity tolerance in chrysanthemum. Plant Cell Rep. 36, 571–581. doi: 10.1007/s00299-017-2103-6, PMID: 28116501

[ref113] Wani S. H. Kumar V. Khare T. Guddimalli R. Parveda M. Solymosi K.. (2020). Engineering salinity tolerance in plants: progress and prospects. Planta 251:76. doi: 10.1007/s00425-020-03366-6, PMID: 32152761

[ref114] Watanabe K. Kobayashi A. Endo M. Sage-Ono K. Toki S. Ono M. (2017). CRISPR/Cas9-mediated mutagenesis of the *dihydroflavonol-4-reductase-B (DFR-B)* locus in the Japanese morning glory *ipomoea (Pharbitis) nil*. Sci. Rep. 7:10028. doi: 10.1038/s41598-017-10715-1, PMID: 28855641 PMC5577235

[ref115] Wu S. Sun Y. Niu G. (2016). Morphological and physiological responses of nine ornamental species to saline irrigation water. HortScience 51, 285–290. doi: 10.21273/HORTSCI.51.3.285

[ref116] Xu J. Kang B. C. Naing A. H. Bae S. J. Kim J. S. Kim H.. (2020). CRISPR/Cas9-mediated editing of 1-aminocyclopropane-1-carboxylate oxidase1 enhances petunia flower longevity. Plant Biotechnol. J. 18, 287–297. doi: 10.1111/pbi.13197, PMID: 31222853 PMC6920161

[ref117] Yan R. Wang Z. Ren Y. Li H. Liu N. Sun H. (2019). Establishment of efficient genetic transformation systems and application of CRISPR/Cas9 genome editing technology in *Lilium pumilum* DC. Fisch. and *Lilium longiflorum* white heaven. Int. J. Mol. Sci. 20:2920. doi: 10.3390/ijms20122920, PMID: 31207994 PMC6627044

[ref118] Yang R. Chen M. Sun J.-C. Yu Y. Min D.-H. Chen J.. (2019). Genome-wide analysis of LIM family genes in foxtail millet (*Setaria italica* L.) and characterization of the role of *SiWLIM2b* in drought tolerance. Int. J. Mol. Sci. 20:1303. doi: 10.3390/ijms20061303, PMID: 30875867 PMC6470693

[ref119] Yang Y. Guo Y. Zhong J. Zhang T. Li D. Ba T.. (2020). Root physiological traits and transcriptome analyses reveal that root zone water retention confers drought tolerance to *Opisthopappus taihangensis*. Sci. Rep. 10:2627. doi: 10.1038/s41598-020-59399-0, PMID: 32060321 PMC7021704

[ref120] Yang Z. Wang Y. Wei X. Zhao X. Wang B. Sui N. (2017). Transcription profiles of genes related to hormonal regulations under salt stress in sweet sorghum. Plant Mol. Biol. Report. 35, 586–599. doi: 10.1007/s11105-017-1047-x

[ref121] Yue Y. Zhang M. Zhang J. Duan L. Li Z. (2012). *SOS1* gene overexpression increased salt tolerance in transgenic tobacco by maintaining a higher K^+^/Na^+^ ratio. J. Plant Physiol. 169, 255–261. doi: 10.1016/j.jplph.2011.10.007, PMID: 22115741

[ref122] Zandalinas S. I. Mittler R. Balfagón D. Arbona V. Gómez-Cadenas A. (2018). Plant adaptations to the combination of drought and high temperatures. Physiol. Plant. 162, 2–12. doi: 10.1111/ppl.12540, PMID: 28042678

[ref123] Zeng Y. Li Q. Wang H. Zhang J. Du J. Feng H.. (2018). Two NHX-type transporters from *Helianthus tuberosus* improve the tolerance of rice to salinity and nutrient deficiency stress. Plant Biotechnol. J. 16, 310–321. doi: 10.1111/pbi.12773, PMID: 28627026 PMC5785360

[ref124] Zhang B. Xie L. Sun T. Ding B. Li Y. Zhang Y. (2019). Chrysanthemum morifolium aquaporin genes CmPIP1 and CmPIP2 are involved in tolerance to salt stress. Sci. Hortic. 256:108627. doi: 10.1016/j.scienta.2019.108627

[ref125] Zhao Z. Li T. Cheng Y. Wang F. Zhao X. (2021). Morphological and metabolic responses of four *Iris germanica* cultivars under salinity stress. Sci. Hortic. 281:109960. doi: 10.1016/j.scienta.2021.109960

[ref126] Zhao D. Tao J. (2015). Recent advances on the development and regulation of flower color in ornamental plants. Front. Plant Sci. 6:261. doi: 10.3389/fpls.2015.00261, PMID: 25964787 PMC4410614

[ref127] Zhao C. Zhang H. Song C. Zhu J.-K. Shabala S. (2020). Mechanisms of plant responses and adaptation to soil salinity. Innovation 1:100017. doi: 10.1016/j.xinn.2020.100017, PMID: 34557705 PMC8454569

[ref128] Zheng T. Li P. Li L. Zhang Q. (2021). Research advances in and prospects of ornamental plant genomics. Hortic. Res. 8:65. doi: 10.1038/s41438-021-00499-x, PMID: 33790259 PMC8012582

[ref129] Zhu J. K. (2003). Regulation of ion homeostasis under salt stress. Curr. Opin. Cell Biol. 6, 441–445. doi: 10.1016/S1369-5266(03)00085-2, PMID: 12972044

