# Supplementary figures and images for: Mechanisms of Salt Tolerance and Molecular Breeding of Salt-Tolerant Ornamental Plants
Source: Front Plant Sci. 2022 Apr 27;13:854116. doi: 10.3389/fpls.2022.854116 (PMC9093713; doi:10.3389/fpls.2022.854116)

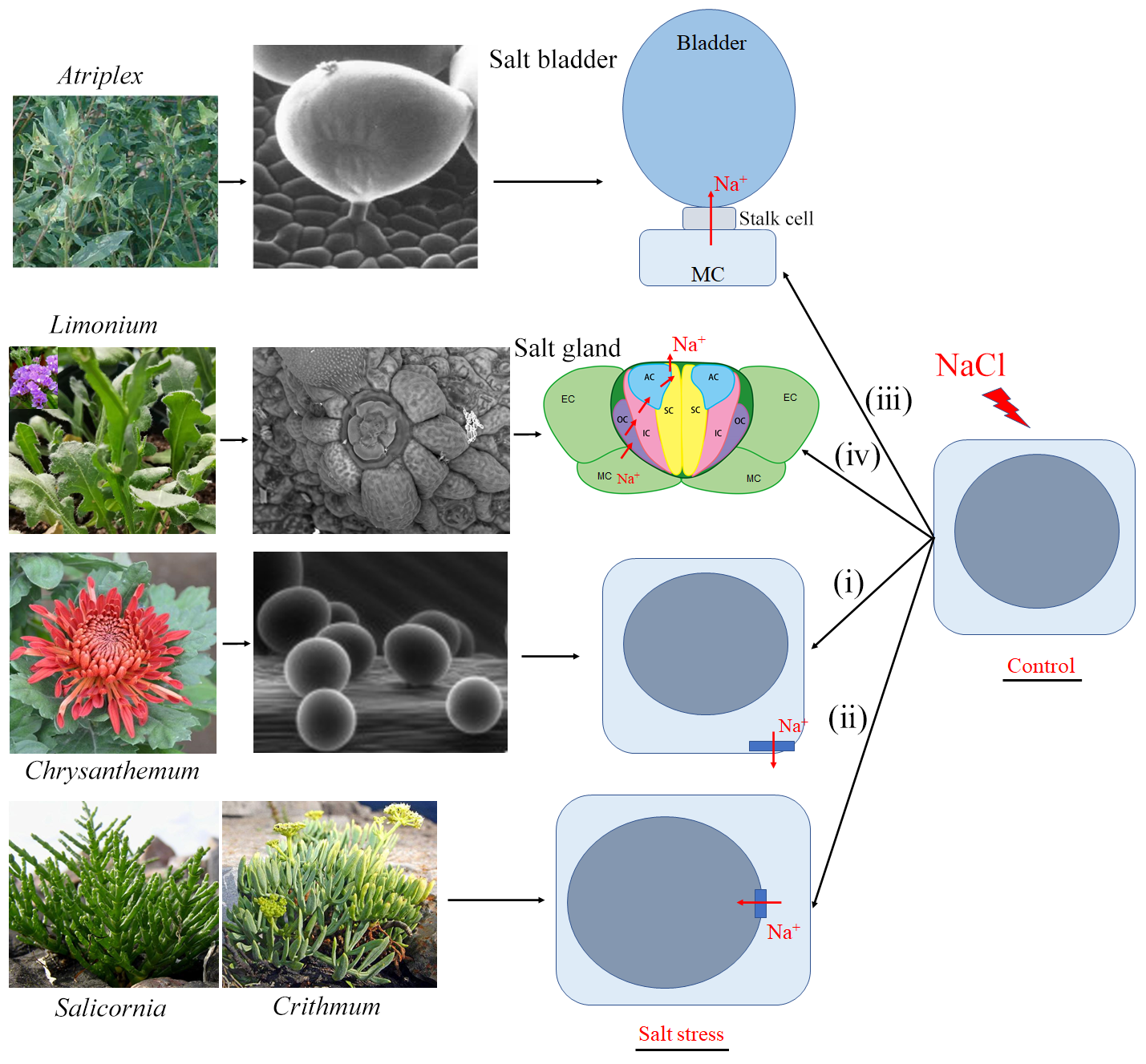

Supplement: Supplementary Figure S1 — Summarized structural adaptation to salinity of halophytic ornamental plants. Notes: (i) pumping Na+ back to the environment; (ii) depositing large amounts of salt ions in vacuoles of succulent tissues; (iii) secreting salt ions through salt bladder; (iv) secreting salt ions through salt glands. MC, mesophyll cell; EC, epidermal cell; OC, outer cup cell; IC, inner cup cell; AC, accessory cell; and SC, secretory cell. [file Image_1.TIF]
