# Supplementary material for: Mechanisms of Salt Tolerance and Molecular Breeding of Salt-Tolerant Ornamental Plants
Source: Front Plant Sci. 2022 Apr 27;13:854116. doi: 10.3389/fpls.2022.854116 (PMC9093713; doi:10.3389/fpls.2022.854116)
Supplement: Supplementary file 2 [file Table_1.DOC]

**TABLE S1** Varieties with enhanced salinity tolerance by genetic technology

| **Species** | **Methods / techniques** | **Main Phenotype** | **References** |
| --- | --- | --- | --- |
| *Chrysanthemum*  *crassum* | Overexpressing *CcSOS1* genes | Reduced Na^+^ content and maintained a favorable K^+^/Na^+^ ratio | An et al., 2014 |
| *Dendronthema grandiform* | Overexpressing *DgNAC1* gene | Enhanced salt tolerance with higher activities of SOD, POD, CAT compared with wild type | Wang et al., 2017b |
| *Dendronthema grandiform* | Overexpressing *DgWRKY4* gene | Enhanced salt tolerance with higher activities of SOD, POD, CAT compared with wild type | Wang et al., 2017a; |
| *Chrysanthemum morifolium* | Overexpressing *CmPIP1* and *CmPIP2* genes | Enhanced salt tolerance with reduced malondialdehyde content | Zhang et al., 2019 |
| *Ipomoea batatas* | Overexpressing *IbNHX2* gene | Enhanced salt and drought tolerance | Wang et al., 2016 |
| *Osmanthus fragrans* | Gamma irradiation | Enhanced salt tolerance and osmotic regulation ability, greater ROS scavenging capacity | Geng et al., 2019 |
